# Integration of QTL Mapping and Gene Fishing Techniques to Dissect the Multi-Main Stem Trait in Rapeseed (*Brassica napus* L.)

**DOI:** 10.3389/fpls.2019.01152

**Published:** 2019-09-20

**Authors:** Weiguo Zhao, Hongbo Chao, Lina Zhang, Na Ta, Yajun Zhao, Baojun Li, Kai Zhang, Zhoubo Guan, Dalin Hou, Kang Chen, Huaixin Li, Libin Zhang, Hao Wang, Maoteng Li

**Affiliations:** ^1^Department of Biotechnology, College of Life Science and Technology, Huazhong University of Science and Technology, Wuhan, China; ^2^Hybrid Rape Research Center of Shaanxi Province, Shaanxi Rapeseed Branch of National Centre for Oil Crops Genetic Improvement, Yangling, China

**Keywords:** *Brassica napus*, multi-main stem trait, QTL mapping, Gene-Fishing, candidate genes

## Abstract

Rapeseed is one of the most important oilseed crops in the world. Improving the production of rapeseed is beneficial to relieve the shortage of edible vegetable oil. As the organ of support and transport, the main stem of rapeseed controls the plant architecture, transports the water and nutrients, and determines the number of inflorescence. Increasing the number of main stems would be helpful for the yield improvement in *Brassica napus* (*B. napus*). This attractive multi-main stem (MMS) trait was observed in the KN DH population. We investigated not only the frequency of MMS traits but also dissected the genetic basis with QTL mapping analysis and Gene-Fishing technique. A total of 43 QTLs were identified for MMS based on high-density linkage map, which explained 2.95–14.9% of the phenotypic variation, among which two environmental stable QTLs (c*qMMS.A3-2 and cqMMS.C3-5*) were identified in winter and semi-winter environments. Epistatic interaction analysis indicated *cqMMS.C3-5* was an important loci for MMS. According to the functional annotation, 159 candidate genes within QTL confidence intervals, corresponding to 148 *Arabidopsis thaliana* (*A. thaliana*) homologous genes, were identified, which regulated lateral bud development and tiller of stem, such as shoot meristemless (*STM*), *WUSCHEL*-regulated-related genes, cytokinin response factors (*CRF5*), cytokinin oxidase (*CKX4*), gibberellin-regulated (*RDK1*), auxin-regulated gene (*ARL*, *IAR4*), and auxin-mediated signaling gene (*STV1*). Based on Gene-Fishing analysis between the natural plants and the double-main stem (DMS) plant, 31 differentially expressed genes (DEGs) were also obtained, which were related to differentiation and formation of lateral buds, biotic stimulus, defense response, drought and salt-stress responses, as well as cold-response functional genes. In addition, by combining the candidate genes in QTL regions with the DEGs that were obtained by Gene-Fishing technique, six common candidate genes (*RPT2A*, *HLR*, *CRK*, *LRR-RLK*, *AGL79*, and *TCTP*) were identified, which might probably be related to the formation of MMS phenotype. The present results not only would give a new insight into the genetic basis underlying the regulation of MMS but also would provide clues for plant architecture breeding in rapeseed.

## Introduction

Plant architecture is a significant agronomic trait that indirectly influence the seed yield by affecting the formation of photosynthetic product and storage of grain-filling substance. Notably, the first “Green Revolution” was one of the great successes, which had improved plant architecture, especially in significant reduction plant height and an increase of the overall seed yield and harvest index (Khush, 2001). Seed yield per unit area is closely related to many plant architecture elements, including yield component, plant height, tiller number, intersection angle of tiller, and leaf shape. Especially in rice and wheat, the increase of the effective tiller number could improve their seed yield per plant and thus increase the overall yield. Some studies that are focused on the mechanism of plant architecture formation have been reported in rice, *Arabidopsis thaliana* (*A. thaliana*) and tomato (Leibfried et al., 2005; Jiang et al., 2013; Zhou et al., 2016). Therefore, improving the plant architecture of crops could effectively increase the crop yield.

Rapeseed (*Brassica napus*, AACC genome, 2n = 38) originated from a spontaneous hybridization between *Brassica rapa* (AA, 2n = 20) and *Brassica oleracea* (CC, 2n = 18) (Nagaharu, 1935). Rapeseed is cultivated throughout the world for the production of vegetable oil, animal feed, and biodiesel. At present, the proportion of rapeseed oil in the total oil production of crop is as high as 57.2% in China (Xie et al., 2005; Yin et al., 2010); in the meanwhile, 60% of edible oil have to be imported from abroad (Wang and Yin, 2014). Seed yield of rapeseed is directly determined by yield-component traits, including seed number per silique, seed weight, and the number of effective siliques per plant (Qzer et al., 1999; Quarrie et al., 2006). Several indirectly related traits, including plant height, the number of the first effective branch, and the silique number of main inflorescence, were also important contributions. The genetic basis of the seed yield components and seed yield–related traits have been dissected by quantitative trait loci (QTL) mapping (Chen et al., 2007; Ding et al., 2012; Zhao et al., 2016) and genome-wide association studies (GWAS) (Liu et al., 2016; Lu et al., 2016).

In recent years, the new agronomic traits, multi-main stem trait (MMS, including double-main stems, three main stems, and over three main stems), have been discovered in rapeseed. MMS is an important seed yield–related trait, which was differentiated from shoot meristem. The plants with MMS have many advantages, such as multiple main stems (or tiller number), higher growth potential, fewer branches, and more seed number, which play an important role for increasing seed yield in rice and wheat (Lu et al., 2015; Shao et al., 2019; Hu et al., 2017). Favorable main stem number can also increase planting density, which contributes to a higher yield per unit area. In the process of differentiation, the morphological structure, physiological status, and endogenous hormonal content of the shoot apical meristem (SAM) undergo substantial change (Gordon et al., 2009). The SAM must maintain a balance between stem cell niches updating and peripheral organ initiation in order to fulfill this function. The genes that cause MMS might have a relationship with *WUSCHEL* (*WUS*) and *CLAVATA* (*CLV*) (Brand et al., 2002; Müller et al., 2006). In normal condition, the gene *WUS* is always expressed in a small group of cells in the organization center of the SAM, underneath the three outer cell layers (Weigel and Jürgens, 2002; Williams and Fletcher, 2005). Cells in center zone proliferate to form two kinds of cells (Haecker and Laux, 2001): one is progeny of stem cell holding multipotency, which maintain the structure of center zone, and the other is a daughter cell, who will develop into perimeter zone and then differentiate into various organizations. Only when the two progresses develop at the same pace, SAM will develop into normal structure; otherwise, the SAM will abnormally develop (Wang and Li, 2008). During development process of SAM, *WUS* functions in maintaining the stem cells undifferentiated to keep natural structure and function of SAM (Schoof et al., 2000). Loss of function of *WUS* would lead to a large amount of stem cells developed into perimeter zone, and then giving rise to the leaf primordiums and secondary SAMs that will come into being (Wang and Li, 2008). Another important gene in SAM is *CLV* that can promote stem cells to develop into perimeter zone. A *WUS-CLV* feedback loop in plant exists, which is of great importance to maintain normal structure of SAM. C*LV3* encodes a kind of secreted protein to activate *CLV1/CLV2* complex when the number of stem cells in SAM increase. When *WUS* is inhibited, the number of stem cells will decrease which in turn restrains the expression of *CLV3* (Fletcher and Meyerowitz, 2000; Dodsworth, 2009). Based on the previous reports, the maintenance homeostasis of SAM mainly includes *WUS-CLV* feedback loop (Fletche, 2018), phytohormone (Brand et al., 2000), redox regulation (Schippers et al., 2016), and network consisting of various genes.

QTL mapping has been proved to be an important approach for dissecting complex traits (Paran and Zamir, 2003). Many agricultural traits, for example, seed yield (Zhao et al., 2016), seed yield–related traits (Shi et al., 2009; Zhao et al., 2016), oil content (Wang et al., 2013), and fatty acid composition (Jiang et al., 2014; Bao et al., 2018), have been dissected by QTL mapping. In addition, some important genes that regulated agricultural traits of rapeseed were identified by fine mapping and map-based cloning, such as recessive genic male serility *BNAMS1* (Yi et al., 2006), seed weight *TSWA5a* and *TSWA5c* (Fan et al., 2010), and seed number per silique *BnaC9.SMG7b* (Li et al., 2015), etc. Gene-Fishing technology, based on PCR technology, could identify the differentially expressed genes quickly (Kim et al., 2004). For example, Choi et al. (2008) found 31 differentially expressed fragments in the grapevines inoculated with *Rhizobium* and salicylic acid by using Gene-Fishing (Choi et al., 2008). Junaedi et al. (2007) explored the effects of high allelopathic rice varieties on barnyard grass and discovered nine differentially expressed fragments (Junaedi et al., 2007). Park et al. (2006) screened the related genes that regulated seed epidermal needling of carrot, and 11 differentially expressed fragments were identified (Park et al., 2006). In addition, some other genes, including *HIP1*, *CSH1*, *PPTSPO1*, and *SLFN-2*, were also identified by Gene-Fishing technique (Cho et al., 2006; Bradley et al., 2007; Frank et al., 2007; Sohn et al., 2007).

In the present study, the MMS characteristics were investigated in a doubled-haploid population named as KN DH population. We counted the number of multi-main stem in each DH line in Yangling and Wuhan for two successive years. Based on QTL mapping and Gene-Fishing technique, the purposes of the present study focused on two aspects: (1) identify QTLs for MMS and obtain candidate genes within QTL regions (2) based on differentially expressed genes and candidate gene within QTL regions and identify several common candidate genes that regulated the MMS.

## Materials and Methods

### Plant Materials and Field Experimental Design in Two Different Ecological Regions

**Figure 1:**
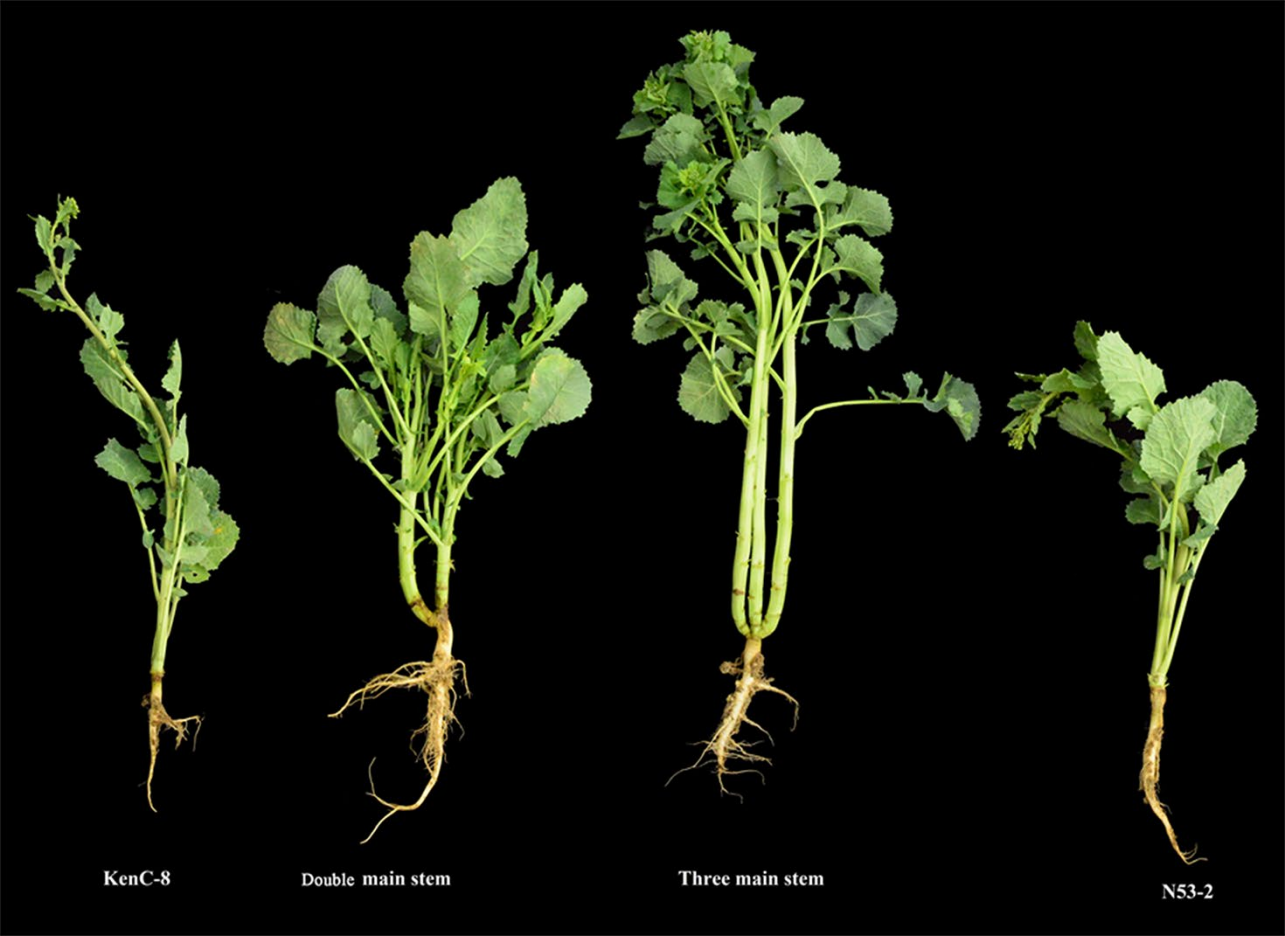
The phenotype of the multi-main stem and their parents in KN population. The parents of KN population include the male parent KenC-8 and the female parent N53-2. The double-main stem and three main stems are located in the KN population.

The KN doubled-haploid (DH) population were derived from bi-parental segregating populations by microspore culture (**Figure 1**) (Wang et al., 2013). The KN population and its parents were sown in the middle of September in the experimental field of Hybrid Rape Research Center of Shaanxi Province, Yangling of Shaanxi province (YL) in northwest China (34°16’N, 108°5’E), and in the early October in the experimental field of Huazhong Agriculture University, Wuhan of Hubei province (WH) in central China (30°47’N, 114°35’E), for two consecutive years from the years 2014 to 2015, respectively. Yangling belonged to the winter ecological region, and Wuhan belonged to the semi-winter ecological region. The KN population and their two parents were planted in Yangling and Wuhan with two replications, respectively. Meanwhile, each particular combination of experimental year × location was defined as an independent experiment. Each independent experiment consisted of 348 DH lines. Each line was grown in a two-row plot with 40 cm between rows and 20 cm between individuals, and row length of 250 cm in all independent experiments. The field experiments followed a randomized complete block design and the normal agricultural practice.

### Phenotypic Statistics and Analysis of MMS

The total number of each line corresponding to the number of plant with MMS in each line was investigated, and the ratio of the MMS was calculated in Yangling of Shaanxi province and Wuhan of Hubei province for two consecutive years. The ratio of the MMS was calculated as the ratio of the number of the MMS to the total number of each line. Not all of the DH lines had MMS in the KN population. In addition, the variance analysis and broad-sense heritability (h2) was calculated by using SAS 8 (SAS Institute Inc, 2000). The formula was h2 = б2g/(б2g + б2ge/n+ б2e/nr) × 100%, б2g is the variance among DH lines, б2ge is the interaction variance of the genotype with environment, б2e is the error variance, n is the number of environments, and r is the number of replications. Meanwhile, a DH lines with double-main stem (DMS) in KN DH population were selected for germination and morphological observation. The early morphology was observed in the five growth stages of seedling stage (15, 25, 30, 35, and 50 days). The seedlings for early morphological observation of the DMS materials and their natural plant type were grown in the culture chamber with 16 h light per day at 22°C ± 4°C.

### QTL Mapping and Identification of Candidate Genes

The ratio of MMS in the KN population was collected as phenotype and used for QTL mapping. A high-density genetic linkage map of the KN population with 3,207 markers, including single-nucleotide polymorphism (SNP), SSR (simple sequence repeat), STS (sequence-tagged site), SRAP (sequence-related amplified polymorphism), and IFLP (intron fragment length polymorphism) markers, was constructed. The total length reached 3072.7 cM, and the average distance was only 0.96 cM between adjacent markers (Chao et al., 2017). Combining the ratio data of MMS with the high-density linkage map of KN population, QTL detection for MMS was performed using a composite interval mapping (CIM) with Windows QTL Cartographer 2.5 software (Wang et al., 2007). The scan walking speed was 1 cM; the window size was 10 cM with five background cofactors. According to the 1,000-permutation test corresponding to *P* = 0.05, the LOD value 2.0 was used as threshold for detection of significant QTLs. All detected QTLs were denoted as significant identified QTLs (Burns et al., 2003). The method for QTL nomenclature was as described by Wang et al. (2013). QTL integration was by meta-analysis using BioMercator 4.1 (Arcade et al., 2004). One QTL that was detected in at least two environments was taken to be a stable QTL, otherwise a specific QTL.

The alignment of the genetic map to the physical map and the identification of candidate genes were the same as those described by Chao et al. (2017). According to the collinearity of the high-density genetic map and *B. napus* “*Darmor-bzh*” reference genome (Chalhoub et al., 2014), genome regions corresponding to the QTL confidence intervals (CIs) were identified by using closely linked SNP within QTL CIs. The candidate genes within QTL CIs were regarded as candidate genes (Shi et al., 2015). The orthologs of *A. thaliana* involving SAM were gathered from previous reported (Wang and Li, 2008). The orthologous of candidate genes and their annotation were obtained by BLASTn based on *A. thaliana* database (http://www.arabidopsis.org/).

### Additive × Additive Epistatic Interactions for MMS

To estimate the epistasis interaction of MMS, we used inclusive ICIM method with IciMapping software to analyze epistatic loci pairs (Li et al., 2008; Meng et al., 2015). To identify significant epistasis interaction loci, a walking speed was set to 1 cM, and significant LOD threshold was set at the same as default parameter of 5.0. The epistasis interaction loci were defined by abbreviation “Ep” of epistasis and trait name and chromosome number as well as location on chromosome (e.g., *EpMMS.A8-115*).

### Acquisition of Differential Expression Genes Based on Gene-Fishing™ Technology

Differential expression genes (DEGs) were identified by using Gene-Fishing^™^ DEG Premix Kits (Seegene, Seoul, South Korea), with an annealing control primer (ACP)–based PCR method (Kim et al., 2004). The total RNA of the DMSs was extracted and then reversed transcription for cDNA by using ReverTra Ace-a-(code no. FSK-100, TOYOBO). According to Gene-Fishing^™^ PCR liquid system (20µl total volume system included cDNA 5µl, arbitrary ACP [5 µM] 2µl, dT-ACP2 [10 µM] 1µl, distilled water 2µl, 2×SeeAmp^™^ACP^™^Master Mix 10µl). The amplified PCR products were separated by 2% agarose gels electrophoresis with ethidium bromide, and differential expression fragments were discriminated. Differential expression fragments were isolated by using AxyPrep DNA Gel Extraction Kit (TIANGEN) and connected to a pMD 18-T Simple Vector (TaKaRa) and were sequenced. Sequencing data were confirmed with the GenBank database through the BlastX program of NCBI (http://www.ncbi.nlm.nih.gov/BLAST/) and/or the *A. thaliana* and *B. napus* database. Then, the sequence differences of genes were compared in natural plant and the DMS plant.

### Quantitative Real-Time PCR (qPCR) Analysis of Six Potential Candidate Genes Regulating MMS

In order to investigate the potential candidate genes regulating MMS, several common candidate genes were selected for quantitative real-time PCR analysis. The shoot meristem of the natural plant and DMS plant in 5, 10, 15, and 20 days after germination was collected and used for relative expression analysis. The total RNA extraction and reversed transcription were the same as above method. Gene-specific primers were designed by using Primer 5.0, and the primers of the six target genes and reference gene actin were listed in **Table S1**. The RT-PCR reaction system was performed with three technical replicates by using TOYOBO SYBR^®^ R Green Realtime PCR Master Mix (code no. QPK-201) Kit. The amplification program was as follow: 95°C for 10 min, then 40 cycles with 95°C for 15s, 60°C for 15 s, and 72°C for 30 s, the last 60 to 95°C to do the melting curve.

## Results

### Phenotype Investigation of KN DH Population and Performance of Multiple Main Stem

**Figure 2:**
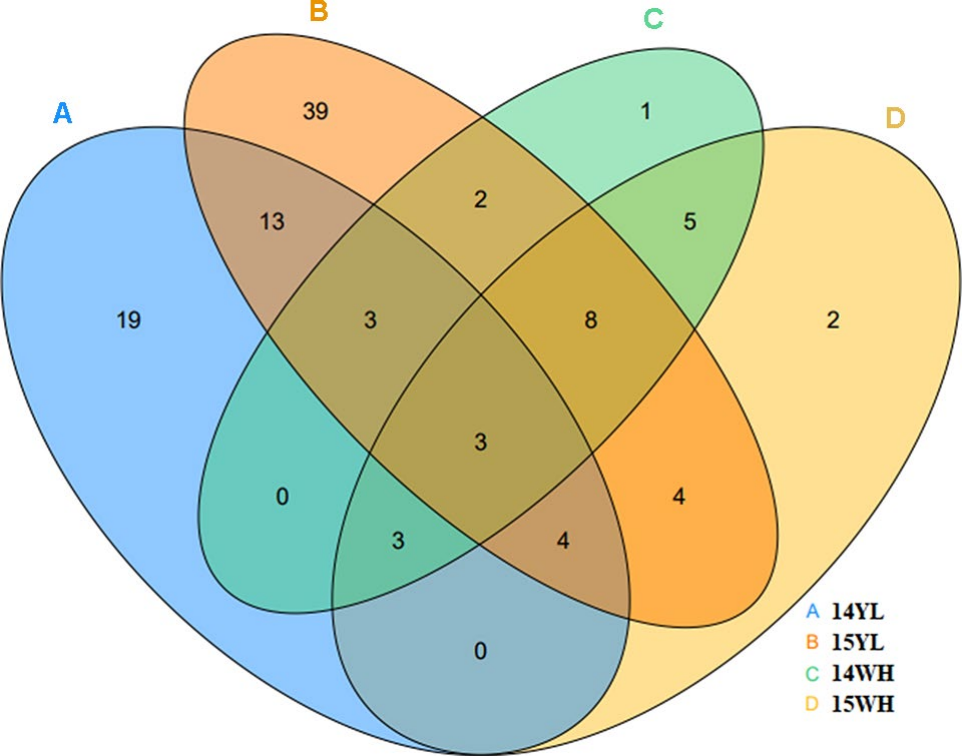
The number of multi-main stem in in different plant environments. **(A)** represents the number of multi-main stem in 2014 in Yangling of Shaanxi province. **(B)** represents the number of multi-main stem in 2015 in Yangling of Shaanxi province. **(C)** represents the number of multi-main stem in 2014 in Wuhan of Hubei province. **(D)** represented the number of multi-main stem in 2015 in Wuhan of Hubei province.

We investigated the plant with MMS for each line in the KN DH population for two consecutive years from the years 2014 to 2015 in Wuhan and Yangling. According to investigations in the years 2014 and 2015, 45 and 76 DH lines showed MMS phenotype in Yangling; however, only 25 and 29 DH lines showed MMS phenotype in Wuhan (**Figure 2**, **Table 1**). Only three lines (QT22, QT229, and QT236) of the 348 lines were found with MMS in all four environments. According to the number of plant with MMS and the total number of each line, the ratio of the MMS occurrence was calculated, the highest ratio of MMS was 100% in Yangling in the years 2014 and 2015, and in Wuhan in the year 2015, the lowest ratio was 20.1% in the year 2015 in Yangling (**Table 1**). In addition, 7.18–21.84% of plants in KN population had the phenotypes of MMS (from two to three main stems), with more leaves in comparison to their parents (**Figure 1**, **Table 1**). Furthermore, broad-sense heritability (*h^2^*) was also calculated for MMS (**Table S2**). MMS had an *h^2^* value 62.26% in Yangling, and an *h^2^* value of 70.23% in Wuhan; these results indicated that the MMS had greater sensitivity and plasticity and was easily influenced by environmental condition.

**Table 1:** The multi-main stem phenotypic ratio of KN DH population in Yangling and Wuhan.

Year/location	14 Yangling	15 Yangling	14 Wuhan	15 Wuhan
Mean	47.87%	41.88%	42.41%	47.26%
Min	21.70%	20.10%	20.50%	22.20%
Max	100.00%	100.00%	94.30%	100.00%
MMS lines	45	76	25	29
Total lines	348	348	348	348
MMS ratio	12.93%	21.84%	7.18%	8.33%
*h* ^2^	62.26%		70.23%	

MMS, multiple main stem; 14 Yangling and 15 Yangling represent the years 2014 and 2015 in Yangling of Shaanxi province, respectively; 14 Wuhan and 15 Wuhan represent the years 2014 and 2015 in Wuhan of Hubei province; h^2^, broad-sense heritability.

**Figure 3:**
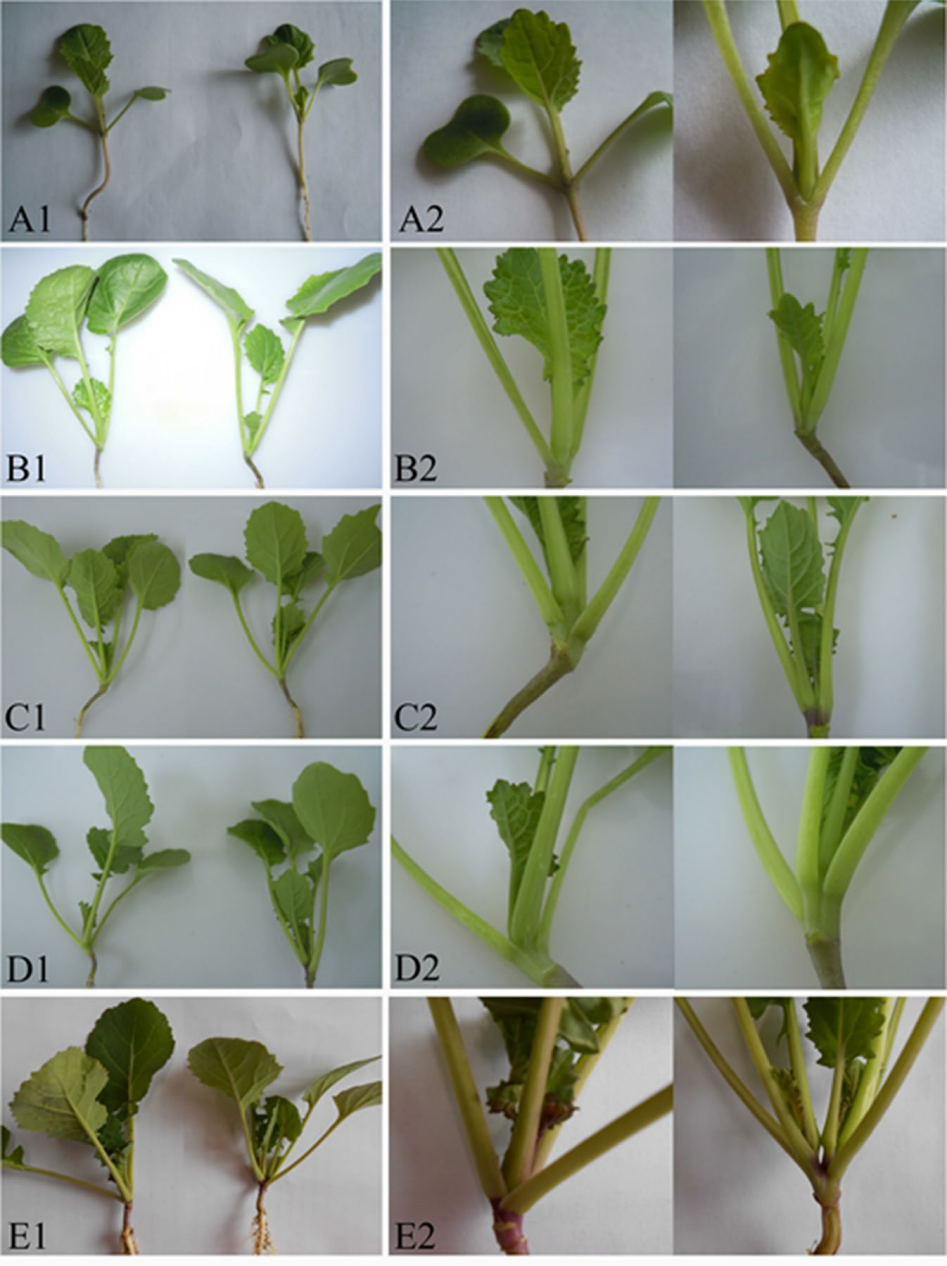
Formation and development of the double-main stem in four seedling stages. **(A1**, **B1**, **C1**, **D1**, and **E1)** on the left represent the natural plant, and **(A1**, **B1**, **C1**, **D1**, and **E1)** on the right represent the double-main stem plant in 15, 25, 30, 35, and 50 days of seedling stages. **(A2**, **B2**, **C2**, **D2**, and **E2)** on the left show enlarge image of the natural plant, and **(A2**, **B2**, **C2**, **D2**, and **E2)** on the right show magnification of **(A1**, **B1**, **C1**, **D1**, and **E1)**.

Moreover, the plant with DMS and the natural normal plants from QT22 were used for germination and morphological development observation in 15, 25, 30, 35, and 50 days of different seedling stages (**Figure 3**). It was revealed that from germination to 25 days, no obvious differences were observed between the DMS plant and the normal plant, and DMS was first observed in 30 days. The stem base of the DMS plant hypertrophied gradually and had formed two main terminal buds. In 35 days, the DMS plant had formed two stems and could be obviously observed. In 50 days, the two main stems of DMS plants developed simultaneously and formed a plant type of DMS. These results indicated that the genes regulating the formation of the DMS function was in early developmental stages. The DMS plants had also grown more leaves and fewer branches compared to the natural plant (**Figure 1**, **Figure 3E2**).

### QTL Mapping for MMS in the KN Population and Candidate Genes Identification

**Figure 4:**
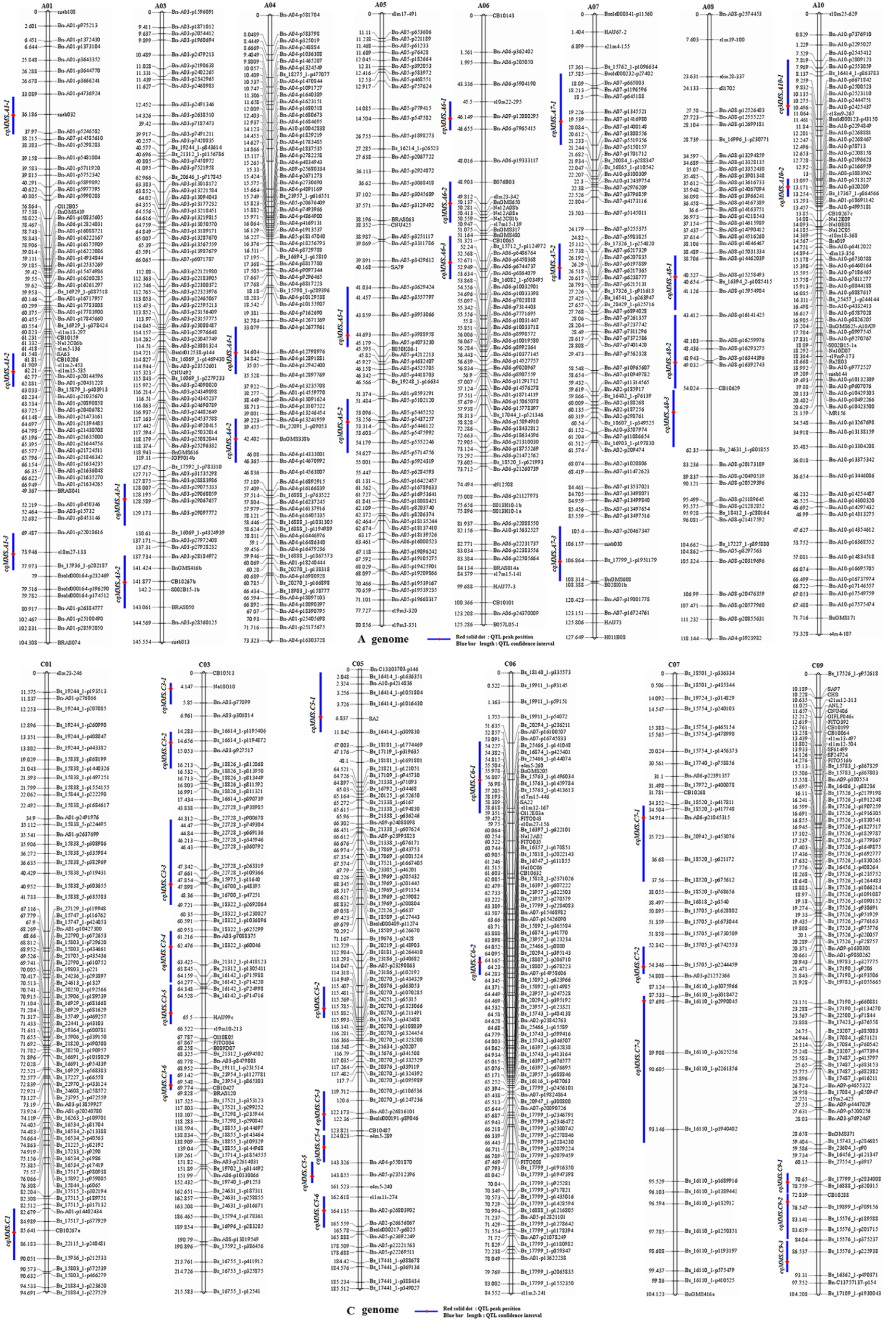
Distribution of QTLs for multi-main stem on the KN high-density map. The 41 consensus QTLs for MMS are distributed on 14 linkage groups with the exception of A02, A09, C02, C04, and C08. : Red solid dot, QTL peak position. Blue bar length, QTL confidence interval.

**Figure 5:**
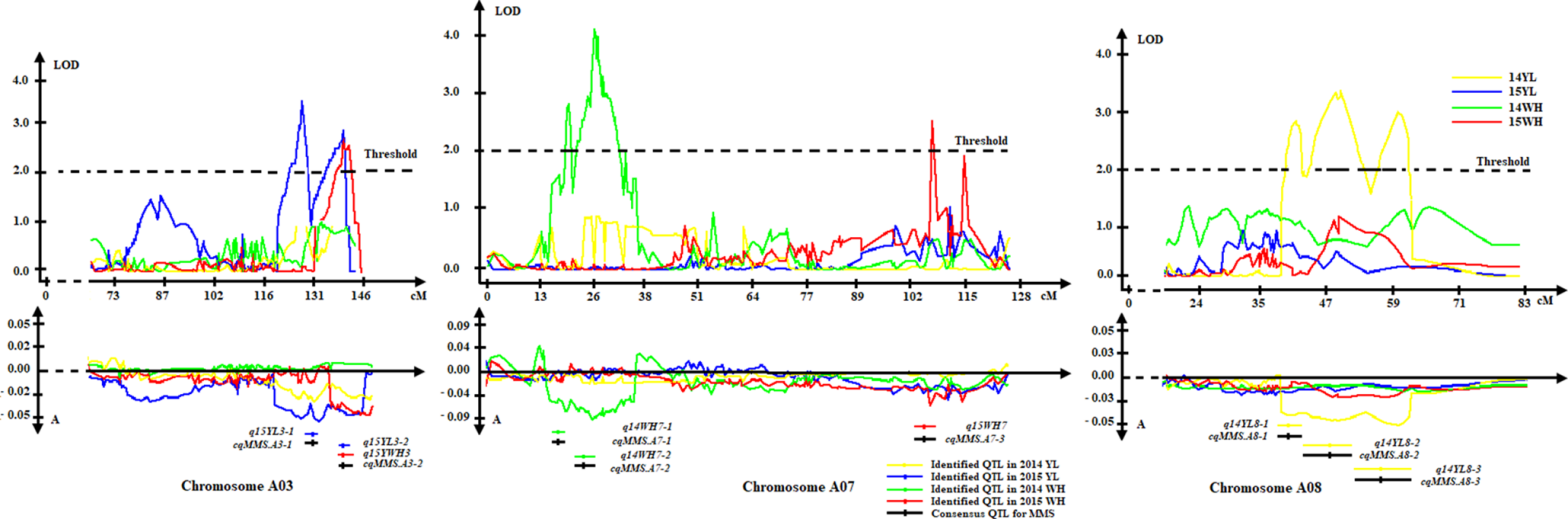
Environmental stable QTL and environmental specific QTL on A03, A07, and A08 chromosomes. In the left, orange QTL *cqMMSa3-2* represents environmental stable QTL in Wuhan and Yangling. Middle green and red QTLs represent specific QTL in Wuhan. Right yellow QTLs represent specific QTL in Yangling.

The MMS ratio in Yangling and Wuhan of the years 2014 and 2015 was used for QTL identification. Totally, 43 identified QTLs for MMS were detected in the KN DH population, which were located on 14 chromosomes, except for A02, A09, C02, C04, and C08 (**Figure 4**, **Table 2**, **Figure S2-1**, **Figure S2-2**). It was revealed that *q14WH7-2*, *q14WH15-1*, and *q15WH13-2* could explain the phenotypic variation more than 10%, which reached 11.38, 11.42, and 14.9%, respectively. Otherwise, QTL *q14WH10-1* and *q14WH10-2* were with the smallest phenotypic variation of 2.95%. In addition, the additive effects of QTLs were from −0.14 to 0.09, and the average CI was 4.77 cM (**Table 2**). QTLs for MMS with overlapping CIs were integrated into consensus QTLs by using BioMercator 4.2 software. Forty-three identified QTLs were integrated into 41 consensus QTLs, of which 39 consensus QTLs that were only expressed in Yangling or Wuhan, were considered as environmental specific QTLs (**Table 2**, **Figure 4**, **Figure 5**). The remaining two consensus QTLs (c*qMMS.A3-2 and cqMMS.C3-5*) were repeatedly detected in two successive years in Yangling and Wuhan (**Table 2**, **Figure 5**), respectively, which were considered to be environmental stable QTLs. These results indicated that the majority of consensus QTLs for MMS were expressed in a specific environment, and MMS was greatly affected by environmental conditions. Regretfully, no major QTL for MMS was identified in all environments.

**Table 2:** Information of QTLs for multi-main stem in the KN population.

Consensus QTL	Chr	Peak (cM)	CIs (cM)	Range (cM)	Identified QTL	LOD	PV (%)	A	E
cqMMS.A1-1	A01	36.21	33.20–38.00	4.80	q15YL1-1	2.88	4.99	0.06	YL
cqMMS.A1-2	A01	62.91	62.20–63.50	1.30	q15YL1-2	2.38	9.18	0.08	YL
cqMMS.A1-3	A01	74.01	69.50–78.40	8.90	q14YL1	2.12	6.31	0.07	YL
cqMMS.A3-1	A03	128.61	128.00–132.30	2.20	q15YL3-1	3.66	6.05	−0.04	YL
cqMMS.A3-2	A03	141.91	137.70–143.10	5.40	q15YL3-2	2.93	4.88	−0.04	YL
					q15WH3	2.82	5.02	−0.04	WH
cqMMS.A4-1	A04	34.61	33.10–35.50	2.40	q14YL4-1	3.72	5.97	0.06	YL
cqMMS.A4-2	A04	42.41	39.20–46.40	7.20	q14YL4-2	3.34	5.48	0.05	YL
cqMMS.A5-1	A05	44.71	41.00–45.20	4.20	q14YL5-1	3.02	8.89	0.09	YL
cqMMS.A5-2	A05	53.31	51.50–54.60	3.10	q14YL5-2	2.57	7.82	0.09	YL
cqMMS.A6-1	A06	46.21	45.50–46.70	1.20	q14YL6-1	2.92	4.75	0.04	YL
cqMMS.A6-2	A06	49.81	48.90–50.0	1.70	q15YL6	2.52	4.13	0.04	YL
cqMMS.A6-3	A06	52.31	51.20–53.00	1.80	q14YL6-2	3.24	5.20	0.05	YL
cqMMS.A7-1	A07	19.61	17.60–20.60	3.00	q14WH7-1	2.85	7.99	−0.07	WH
cqMMS.A7-2	A07	25.81	24.80–26.30	1.50	q14WH7-2	4.13	11.38	−0.09	WH
cqMMS.A7-3	A07	106.91	104.80–108.30	3.50	q15WH7	2.45	5.90	−0.06	WH
cqMMS.A8-1	A08	40.51	38.60–43.40	4.80	q14YL8-1	2.73	4.41	−0.04	YL
cqMMS.A8-2	A08	49.01	43.40–54.00	10.60	q14YL8-2	3.42	5.49	−0.05	YL
cqMMS.A8-3	A08	59.01	54.00–62.20	8.20	q14YL8-3	3.05	6.88	−0.05	YL
cqMMS.A10-1	A10	9.31	7.50–9.70	2.20	q14WH10-1	2.86	2.95	0.03	WH
cqMMS.A10-2	A10	12.51	12.2–12.90	0.70	q14WH10-2	2.88	2.95	0.03	WH
cqMMS.C1	C01	85.71	82.70–90.10	7.40	q14YL11	2.53	7.39	−0.08	YL
cqMMS.C3-1	C03	4.21	4.00–5.90	1.90	q14YL13-1	3.12	9.18	−0.09	YL
cqMMS.C3-2	C03	14.71	14.30–16.20	1.90	q14YL13-2	3.03	8.92	−0.09	YL
cqMMS.C3-3	C03	47.91	44.30–49.70	5.40	q15WH13-1	2.38	7.32	0.09	WH
cqMMS.C3-4	C03	62.51	61.00–64.10	3.10	q15YL13	2.82	4.68	−0.04	YL
cqMMS.C3-5	C03	65.31	64.20–66.30	2.10	q14YL13-3	2.20	6.35	−0.07	YL
					q15WH13-2	5.17	14.90	−0.14	WH
cqMMS.C3-6	C03	69.61	69.20–69.80	0.60	q15WH13-3	2.53	7.66	−0.10	WH
cqMMS.C5-1	C05	5.71	0.00–13.20	13.20	q14WH15-1	3.86	11.42	0.09	WH
cqMMS.C5-2	C05	115.01	114.10–115.40	1.30	q15WH15-1	3.28	8.86	0.07	WH
cqMMS.C5-3	C05	122.21	119.70–123.80	4.10	q15WH15-2	2.22	6.11	0.06	WH
cqMMS.C5-4	C05	136.01	124.00–143.30	19.30	q15WH15-3	3.03	7.31	0.04	WH
cqMMS.C5-5	C05	143.91	143.30–154.30	11.00	q15WH15-4	2.76	4.93	0.03	WH
cqMMS.C5-6	C05	164.11	162.60–165.60	3.00	q14WH15-2	2.52	6.85	0.07	WH
cqMMS.C6-1	C06	53.11	51.60–54.40	2.80	q14WH16-1	2.29	6.27	−0.07	WH
cqMMS.C6-2	C06	60.31	60.10–60.80	0.70	q14WH16-2	2.15	5.85	−0.07	WH
cqMMS.C7-1	C07	34.91	34.40–37.60	3.20	q15WH17-1	3.08	5.56	−0.03	WH
cqMMS.C7-2	C07	54.41	52.40–54.70	2.30	q15WH17-2	3.11	5.60	−0.03	WH
cqMMS.C7-3	C07	87.71	87.50–94.40	6.90	q14WH17	4.07	11.17	−0.09	WH
cqMMS.C9-1	C09	70.81	65.80–72.80	7.00	q15WH19-1	3.30	9.19	−0.08	WH
cqMMS.C9-2	C09	76.61	72.80–84.00	11.20	q15WH19-2	3.97	10.91	−0.08	WH
cqMMS.C9-3	C09	90.51	84.00–92.50	8.50	q15WH19-3	3.42	10.29	−0.08	WH

Chr, chromosome; CIs, confidence interval; LOD, log odds score; PV, phenotypic variation; A, additive effect; E, environment; YL, Shaanxi Yali; WH, Hubei Wuhan.

According to the co-linearity relationship between the KN high-density genetic map and the *B. napus* reference genome (Chalhoub et al., 2014), 159 genes corresponding to 148 A. *thaliana* homologous genes within CIs of QTLs were identified; these genes were mainly involved in lateral bud development and tiller of stem (**Table 3**, **Table S3**). Both *BnaC09g13580D* (*STM*) and *BnaC09g34650D* (*WOX2*) were located in QTL region of *cqMMS.C9-1*, in which *BnaC09g13580D* participated in stem cell population maintenance (Müller et al., 2006) and cytokinin biosynthetic process (Yanai et al., 2005), and *BnaC09g34650D* belonged to homeobox genes family and mainly regulated the formation and maintenance of SAM (Clark et al., 1996; Laux et al., 1996). *BnaA01g28990D* (*CUC1*) was located in QTL region of c*qMMS.A1-3*, and it maintains boundaries between the SAM and lateral organ primordia (Aida et al., 1999), which was transcriptionally activated by gene *STM* (Spinelli et al., 2011). *BnaA01g28990D* and *BnaC09g13580D* (*STM*) are critical genes in lateral organ differentiation (Aida et al., 1999), of which *STM* promotes cell division by suppressing organ differentiation, and downregulation of *STM* is beneficial to the initiation of lateral organs (Heisler et al., 2005). *BnaA03g50360D* (*BRS1*), *BnaA03g54090D* (*LBD39*), and *BnaA03g54110D* (*NPY5*) were located in stable QTL region of *cqMMS.A3-2* (**Figure 5**). *BRS1* (*BRI1 suppressor 1*) is a serine carboxypeptidase that is involved in brassinosteroid signaling and was recognized to suppress the phenotypes of the brassinosteroid receptor weak mutant bri1-5 (Deng et al., 2017). *NPY5* (*naked pins in yuc mutants 5*) was highly concentrated in cotyledons and was involved in auxin-mediated organogenesis (Cheng et al., 2008). *LBD39* (*LOB domain-containing protein 39*) was a transcription factor in LOB family and could regulate lateral organ boundaries. *BnaA07g02390D* (*WUS*) within QTL region of c*qMMS.A7-1* encodes a homeodomain protein and is expressed at the center of plant meristem, which induces cell proliferation of meristem and maintain the state of meristem (Kieffer et al., 2006; Sablowski, 2007). *BnaC05g48100D* (*WOX11*) was located in QTL region of *qMMS.C5-2*, and it was a transcription factor, which combines with *WOX6* to control the shoot gravitropism and rice tiller angle by regulating asymmetric distribution of auxin (Zhang et al., 2018). *BnaA10g02750D* (*YUC3*) was located in QTL region of *qMMS.A10-1*, which is an important gene involved in auxin biosynthesis (Zhang et al., 2018; Zwack et al., 2016). Moreover, several related genes for cytokinins and auxins were found within QTL regions. *BnaC03g29310D* (*CRF1*) and *BnaC03g25680D* (*CRF5*) were located in c*qMMS-C3-5* and c*qMMS-C3-4* regions, respectively, and *BnaC09g21990D* (*CRF6*) were located in *qMMS.C9-1* region, which were considered to be cytokinin response factors (Zwack et al., 2016; Striberny et al., 2017). *BnaC09g33450D* (*CKX3*) and *BnaC09g36710D* (*CKX7*) located in *qMMS.C9-1* region, and *BnaA03g49660D* (*CKX4*) located in *qMMS.A3-1* were considered to be involved in cytokinin catabolic process (Ding et al., 2015; Köllmer et al., 2014; Choi et al., 2014). In addition, *BnaA06g14040D* (*MP*) and *BnaC05g11390D* (*EPR1*) located in QTL region of *qMMS.A6-2* and *qMMS.C5-1*, respectively, which were considered to be transcription factors and could regulate asymmetric distribution of auxin in upstream of auxin synthesis (Zhang et al., 2018; Krogan et al., 2016).

**Table 3:** Candidate genes in *Brassica napus* and homologous genes in *Arabidopsis thaliana* within QTL regions for MMS.

QTL name	Gene in *B. napus*	Homologous gene in *A. thaliana*	Gene name
cqMMS.A1-1	BnaA01g08980D	AT1G20200	EMB2719
	BnaA01g09110D	AT4G18370	DEG5
	BnaA01g09530D	AT4G18710	BIN2
	BnaA01g09700D	AT4G18890	BEH3
	BnaA01g24810D	AT4G23180	CRK10
cqMMS.A1-2	BnaA01g02910D	AT4G34220	LRR-RLK
	BnaA01g25310D	AT3G21180	ACA9
	BnaA01g24690D	AT3G22440	FLA8
cqMMS.A1-3	BnaA01g27090D	AT1G48620	HON5
	BnaA01g28000D	AT1G51800	
	BnaA01g30930D	AT3G55200	CPSF
	BnaA01g28990D	AT3G15170	CUC1
	BnaA01g28930D	AT3G15500	NAC3
	BnaA01g28500D	AT3G15790	MBD11
	BnaA01g28410D	AT3G15880	WSIP2
	BnaA01g26700D	AT3G18550	BRC1
	BnaA01g07860D	AT4G29040	RPT2A
cqMMS.A3-1	BnaA03g31060D	AT3G08770	ERD10
	BnaA03g51000D	AT3G57230	AGL16
	BnaA03g58880D	AT4G29040	RPT2A
	BnaA03g38750D	AT2G14900	RDK1
	BnaA03g51450D	AT4G31400	CTF7
	BnaA03g51900D	AT4G32040	KNAT5
	BnaA03g52120D	AT4G32551	LUC
	BnaA03g52290D	AT4G32980	ATH1
	BnaA03g52520D	AT4G33430	BAK1
	BnaA03g50910D	AT4G34160	CYCD3
	BnaA03g53400D	AT4G35550	WOX13
	BnaA03g53680D	AT4G37180	UIF1
	BnaA03g49660D	AT4G29740	CKX4
cqMMS.A3-2	BnaA03g50360D	AT4G30610	BRS1
	BnaA03g54090D	AT4G37540	LBD39
	BnaA03g54110D	AT4G37590	NPY5
cqMMS.A4-2	BnaA04g18690D	AT1G05230	HDG2
	BnaA04g12130D	AT2G21330	FBA1
	BnaA04g18030D	AT2G31085	CLE6
	BnaA04g18380D	AT2G31310	LBD14
	BnaA04g27450D	AT3G62040	HAD
cqMMS.A5-1	BnaA05g23180D	AT3G16640	MYB73
	BnaA05g07510D	AT2G36890	RAX2
	BnaA05g07510D	AT2G36890	RAX2
	BnaA05g06540D	AT2G38120	AUX1
	BnaA05g06890D	AT2G37630	AS1
	BnaA05g07740D	AT3G53020	STV1
cqMMS.A5-2	BnaA05g04890D	AT2G45470	FLA8
	BnaA05g09820D	AT2G33835	FES1
	BnaA05g09770D	AT2G33880	HB-3
	BnaA05g09360D	AT2G34440	AGL29
	BnaA05g09150D	AT2G34650	PID
	BnaA05g09120D	AT2G34710	PHB
	BnaA05g09060D	AT2G34780	MEE22
	BnaA05g09010D	AT2G34870	MEE26
	BnaA05g08960D	AT2G34925	CLE42
cqMMS.A6-1	BnaA06g24200D	AT4G01710	CRK
	BnaA06g22000D	AT1G78700	BEH4
	BnaA06g22420D	AT5G62940	HCA2
	BnaA06g06160D	AT1G10370	ERD9
cqMMS.A6-2	BnaA06g30630D	AT3G60900	FLA10
	BnaA06g11350D	AT1G16880	
	BnaA06g14040D	AT1G19850	MP
cqMMS.A6-3	BnaA06g13550D	AT1G67720	
cqMMS.A7-1	BnaA07g02390D	AT2G17950	WUS
cqMMS.A7-2	BnaA07g11450D	AT1G20450	ERD10
	BnaA07g11930D	AT5G67300	MYBR1
	BnaA07g27770D	AT1G69220	SIK1
	BnaA07g28930D	AT1G70830	MLP28
	BnaA07g26650D	AT1G67720	
cqMMS.A8-1	BnaA08g14270D	AT4G26850	VTC2
	BnaA08g15890D	AT4G37930	SHMI
	BnaA08g15900D	AT4G37940	AGL21
	BnaA08g16780D	AT4G39070	BZS1
	BnaA08g16820D	AT4G38970	FBA2
	BnaA08g16720D	AT4G39110	
	BnaA08g16520D	AT4G39400	BRI1
	BnaA08g16380D	AT4G39650	GGT2
	BnaA08g16660D	AT5G10720	HK5
	BnaA08g14480D	AT5G48820	ICK6
cqMMS.A10-1	BnaA10g02750D	AT1G04610	YUC3
	BnaA10g12480D	AT5G59480	HAD
	BnaA10g23990D	AT5G61410	RPE
	BnaA10g29350D	AT5G57950	HLR
cqMMS.C1	BnaC01g21630D	AT3G51030	TRX1
	BnaC01g21860D	AT4G16280	FCA
	BnaC01g22100D	AT4G16110	RR2
	BnaC01g22110D	AT1G27320	HK3
	BnaC01g24570D	AT3G46510	PUB13
	BnaC01g26510D	AT3G50070	CYCD3.3
	BnaC01g26840D	AT1G54990	AXR4
	BnaC01g27460D	AT5G13010	EMB3011
	BnaC01g32740D	AT3G20190	PRK4
	BnaC01g33180D	AT5G60440	AGL62
	BnaC01g35230D	AT3G16640	TCTP
cqMMS.C3-3	BnaC03g13380D	AT5G56600	PRF3
	BnaC03g13120D	AT5G57090	EIR1
cqMMS.C3-4	BnaC03g18890D	AT1G78380	GSTU19
cqMMS.C3-5	BnaC03g25680D	AT2G46310	CRF5
	BnaC03g29310D	AT4G11140	CRF1
	BnaC03g26730D	AT2G44080	ARL
cqMMS.C3-6	BnaC03g20740D	AT2G38120	AUX1
	BnaC03g45610D	AT2G14900	
cqMMS.C5-1	BnaC05g07890D	AT1G10370	ERD9
	BnaC05g11390D	AT1G18330	EPR1
	BnaC05g01780D	AT1G03170	DUF3049
cqMMS.C5-2	BnaC05g46270D	AT3G05530	RPT2A
	BnaC05g02370D	AT1G04250	AXR3
cqMMS.C5-3	BnaC05g46030D	AT3G05800	AtBS1
cqMMS.C5-4	BnaC05g45960D	AT5G48670	AGL80
	BnaC05g45360D	AT2G31530	EMB2289
	BnaC05g23630D	AT1G30610	EMB2279
	BnaC05g48100D	AT3G03660	WOX11
cqMMS.C5-5	BnaC05g47460D	AT3G04100	AGL57
cqMMS.C5-6	BnaC05g46680D	AT3G05120	GID1A
	BnaC05g48320D	AT3G02310	SEP2
cqMMS.C6-1	BnaC06g23160D	AT1G74260	PUR4
	BnaC06g35530D	AT1G74660	MIF1
	BnaC06g21810D	AT1G76520	PILS3
	BnaC06g20740D	AT1G77690	LAX3
	BnaC06g19100D	AT1G80350	ERH3
	BnaC06g19420D	AT1G80680	SAR3
	BnaC06g19460D	AT1G80730	ZFP1
	BnaC06g27630D	AT2G26670	TED4
	BnaC06g20020D	AT3G16830	TPR2
	BnaC06g30400D	AT1G69220	SIK1
	BnaC06g34100D	AT1G73190	TIP3
	BnaC06g28810D	AT1G67720	
	BnaC06g16910D	AT3G58780	SHP1
	BnaC06g24760D	AT4G26000	PEP
cqMMS.C6-2	BnaC06g31170D	AT1G24180	IAR4
	BnaC06g29550D	AT1G68800	TCP12
	BnaC06g30690D	AT1G69500	CYP704B1
	BnaC06g31090D	AT1G69970	CLE26
	BnaC06g31830D	AT1G70700	TIFY7
cqMMS.C7-1	BnaC07g15380D	AT1G20450	ERD10
	BnaC07g16030D	AT5G67300	MYBR1
	BnaC07g23630D	AT3G26520	TIP2
	BnaC07g25970D	AT3G30260	AGL79
	BnaC07g30600D	AT5G23720	PHS1
	BnaC07g26970D	AT5G24860	FPF1
	BnaC07g26450D	AT5G48380	BIR1
cqMMS.C7-3	BnaC03g77490D	AT4G30620	RPT2A
cqMMS.C9-1	BnaC09g32510D	AT1G33390	FAS4
	BnaC09g31940D	AT1G34355	PS1
	BnaC09g13580D	AT1G62360	STM
	BnaC09g21990D	AT3G61630	CRF6
	BnaC09g33500D	AT5G57950	HLR
	BnaC09g34990D	AT3G51550	FER
	BnaC09g32160D	AT5G16560	KAN
	BnaC09g35030D	AT5G19140	AILP1
	BnaC09g36930D	AT5G20930	TSL
	BnaC09g36710D	AT5G21482	CKX7
	BnaC09g36350D	AT5G22650	HD2B
	BnaC09g36060D	AT5G23000	MYB37
	BnaC09g30190D	AT5G53760	MLO11
	BnaC09g31160D	AT5G54770	THI1
	BnaC09g32460D	AT5G55910	D6PK
	BnaC09g32470D	AT5G55920	OLI12
	BnaC09g33450D	AT5G56970	CKX3
	BnaC09g32610D	AT5G57710	SMAX1
	BnaC09g34650D	AT5G59340	WOX2
	BnaC09g34750D	AT5G59370	ACT4

### Additive × Additive Epistatic Interactions for MMS in Different Chromosomes

**Figure 6:**
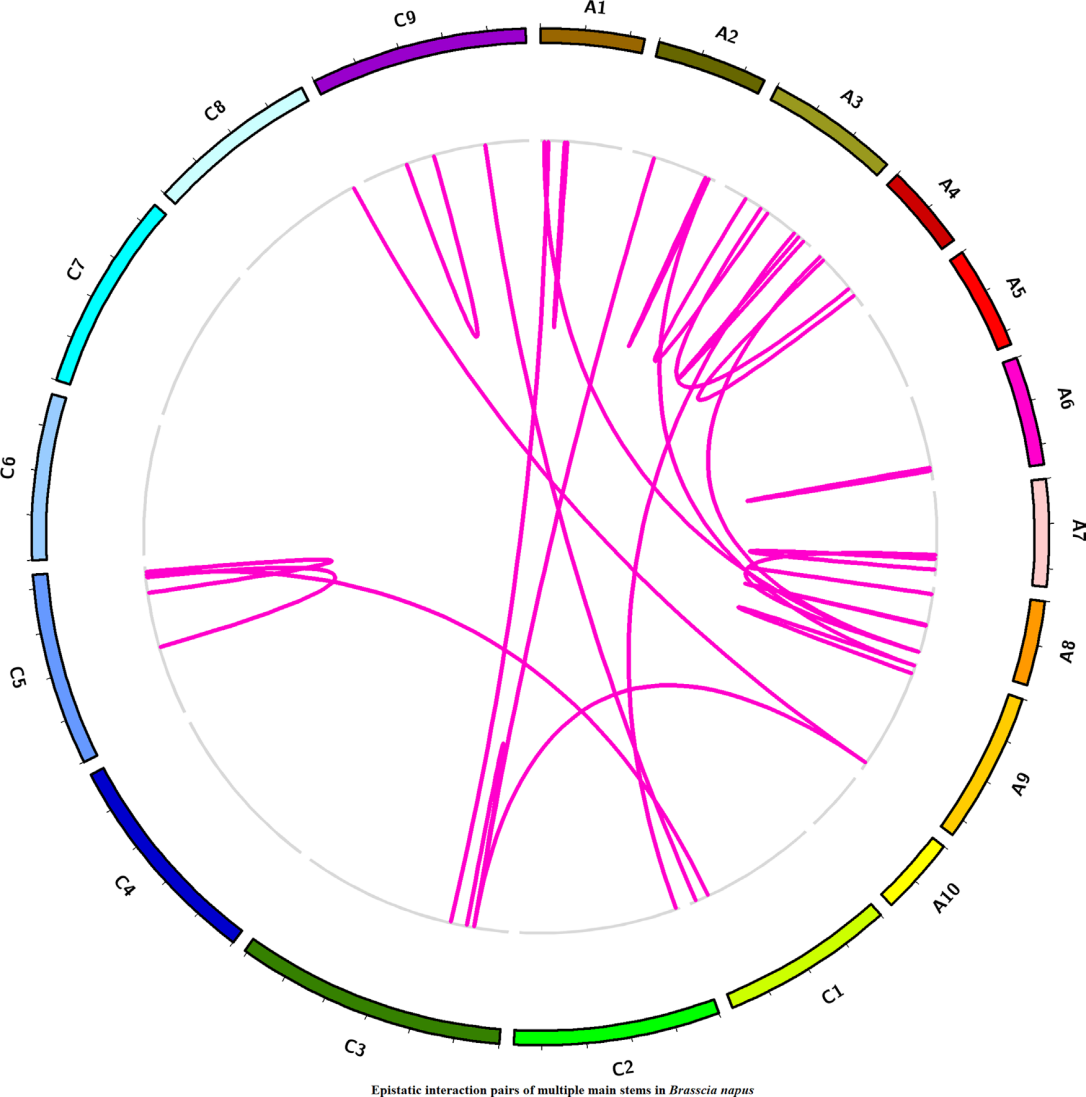
Distribution of epistatic interaction for MMS on different chromosomes. Violet lines represent epistatic interaction pairs of MMS; the epistatic interaction pairs were shown by links in the inside circle. The outside circle represents 19 chromosomes of *Brassica napus*.

To estimate the epistasis interaction for MMS, epistatic loci pairs were detected by using IciMapping software. Totally, 26 statistically significant epistatic loci pairs were detected in three plant regions (**Figure 6**, **Table S4**), which were located on chromosome A01/A08, A01/C03, A02/C03, A02/A09, A03/A04, A04/A08, etc. which accounted for 1.76–17.70% of the phenotypic variance. Five epistatic interaction pairs, including *EpMMS.A3-75*/*EpMMS.A3-25*,* EpMMS.A3-120*/*EpMMS.C2-105*, *EpMMS.A4-5*/*EpMMS.A8-115*, *EpMMS.A9-15*/*EpMMS.A9-160*, and *EpMMS.A9-160*/*EpMMS.C3-65*, had positive epistatic effect, indicating that the effects of parents for MMS were larger than their the recombinant effects of epistatic interaction pairs. The remaining epistatic interaction pairs had negative epistatic effects, indicating that the recombinant effects of these epistatic interaction pairs for MMS were larger than their parental effects. Furthermore, we noted that seven epistatic interaction loci were located in the above-mentioned five QTL intervals. *EpMMS.A7-105* was located in QTL region of *cqMMS.A7-3*, indicating that *cqMMS.A7-3* also interacted with *EpMMS.A7-125*. In addition, three epistatic interaction pairs (*EpMMS.A2-60*/*EpMMS.C3-65*, *EpMMS.C3-65*/*EpMMS.C3-75*, and *EpMMS.A9-160*/*EpMMS.C3-65*) were closely related to *cqMMS.C3-5*, which indicated *cqMMS.C3-5* was an important loci for MMS.

### Differentially Expressed Genes Between the Natural Plant and the DMS Plant by Gene-Fishing™ Technique

**Figure 7:**
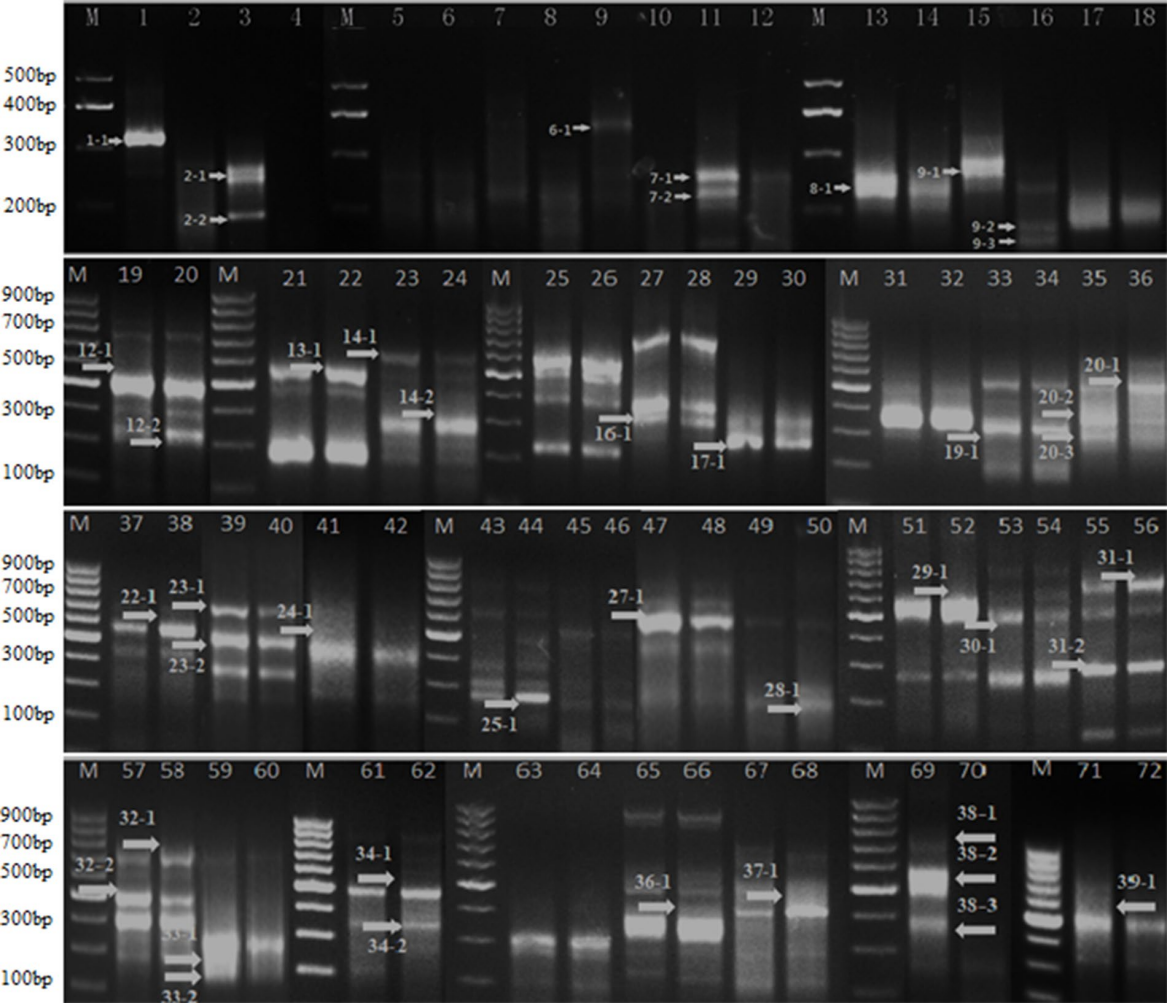
Differential expressed genes screen using ACPs primers based on Gene-Fishing. The numbers and arrows on image represent different differential expression genes. For better presentation, only cropped images of blots are shown here; a full scan of the entire original gels is submitted in the **Supplementary Material** (**Figures S1-1–S1-6**).

Total RNA of main stem apex of the natural plant and DMS in seedling of 20 days were extracted by using Invitrogen TRIzol Kit. The dT-ACP1 primers from Gene-Fishing technique were used as primers for reverse transcription. In order to identify differentially expressed genes (DEGs) in DMS and the natural plant of *B. napus*, the PCR amplification of cDNA using 36 random amplified primers was conducted. Totally, 46 differential expression bands were found, including 28 down-regulated and 18 up-regulated in the DMS compared to the natural plant (**Figure 7**, and a full scan of the entire original gels attached as **Figures S1-1–Figure S1-6** **;** **Table S5**). According to the sequence alignment results, 31 genes were obtained (**Table 4**). Based on functional annotation, seven underlying genes (DEG1, DEG2, DEG4, DEG5, DEG34, DEG36, and DEG46) were annotated to regulate the apical meristem, which might be related to the formation of DMS phenotypes (**Table 4**). In addition, some genes that are related to biotic stimulus, defense response, drought, and salt-stress responses, as well as cold-response functional genes were also identified (**Table 4**).

**Table 4:** Homology comparison of all differential expression genes and corresponding to *Brassica napus* genes and *Arabidopsis thaliana* genes.

DEG number	Score	E-value	Max ident	Gene ID in *B. napus*	Chromosome	*A. thaliana*
DEG1	372	3.00E−99	99.00%	BnaA01g07860D	A01	AT4G29040
				BnaA08g13550D	A08	
				BnaC01g09460D	C01	
				BnaC07g41720D	C07	
				BnaC08g13300D	C08	
DEG2	811	0.00E+00	97.00%	BnaA10g29350D	A10	AT5G57950
				BnaC09g33500D	C09	
DEG3	457	1.00E−124	99.00%	BnaA01g02490D	A01	AT4G34670
				BnaA03g28830D	A03	
				BnaA03g52970D	A03	
				BnaA08g11040D	A08	
				BnaC01g03750D	C01	
				BnaC01g39890D	C01	
				BnaC03g33960D	C03	
				BnaC03g65890D	C03	
				BnaC05g47000D	C05	
				BnaC07g45170D	C07	
DEG4	555	4.00E−154	100.00%	BnaA06g24200D	A06	AT4G01710
DEG5	468	5.00E−128	100.00%	BnaA01g02910D	A01	AT4G34220
				BnaC01g04180D	C01	
DEG6	737	0.00E+00	99.00%	BnaA07g11450D	A07	AT1G20450
				BnaC05g15780D	C05	
				BnaC07g15380D	C07	
DEG8	697	0.00E+00	99.00%	BnaA07g11930D	A07	AT5G67300
				BnaC02g16640D	C02	
				BnaC07g16030D	C07	
DEG9	503	2.00E−138	96.00%	BnaA01g00540D	A01	AT4G37410
				BnaA08g15660D	A08	
				BnaC01g01530D	C01	
				BnaC03g61420D	C03	
DEG10	797	0.00E+00	99.00%	BnaA03g21640D	A03	AT2G47130
				BnaA09g42190D	A09	
				BnaC03g72480D	C03	
				BnaC08g34620D	C08	
DEG11	1177	0.00E+00	100.00%	BnaA03g00940D	A08	AT5G03560
				BnaC03g01290D	C03	
DEG12	736	0.00E+00	99.00%	BnaA02g15190D	A02	AT1G70830
				BnaA07g28930D	A07	
				BnaA08g20650D	A08	
				BnaC02g20340D	C02	
				BnaC06g31980D	C06	
				BnaC07g13200D	C07	
DEG14	503	2.00E−138	94.00%	BnaC01g07930D	C01	AT4G30450
DEG15	460	8.00E−26	96.00%	BnaA01g30930D	A01	AT3G55200
				BnaA09g05750D	A09	
DEG17	1050	0.00E+00	98.00%	BnaA06g05510D	A06	AT1G09690
				BnaA08g26210D	A08	
				BnaA09g48320D	A09	
				BnaC05g07130D	C05	
				BnaC08g13710D	C08	
DEG20	778	0.00E+00	99.00%	BnaA02g24900D	A02	AT5G47030
				BnaA06g35640D	A06	
				BnaC04g00050D	C04	
				BnaC02g33050D	C02	
				BnaC07g19840D	C07	
				BnaC08g25530D	C08	
				BnaC09g19920D	C09	
DEG21	342	2.00E−90	99.00%	BnaA06g06120D	A06	AT1G10330
				BnaA06g06150D	A06	
				BnaC05g07870D	C05	
DEG22	806	0.00E+00	99.00%	BnaA03g17080D	A03	AT2G37220
				BnaA04g04760D	A04	
				BnaA05g07240D	A05	
				BnaC02g22410D	C02	
				BnaC04g08050D	C04	
				BnaC06g04460D	C06	
				BnaC09g41150D	C09	
DEG23	630	8.00E−77	99.00%	BnaA01g26080D	A01	AT2G14900
				BnaA03g38750D	A03	
				BnaC03g40940D	C03	
				BnaC03g45610D	C03	
DEG25	497	6.00E−37	99.00%	BnaA04g17660D	A04	AT2G30570
				BnaA05g11890D	A05	
				BnaC04g13890D	C04	
				BnaC04g41210D	C04	
				BnaC05g04700D	C05	
				BnaC09g18450D	C09	
DEG26	942	0.00E+00	99.00%	BnaA07g29960D	A07	AT1G72230
				BnaC02g21230D	C02	
				BnaC06g33270D	C06	
DEG27	782	0.00E+00	98.00%	BnaA02g28130D	A02	AT3G26520
				BnaA06g32840D	A06	
				BnaC02g36210D	C02	
				BnaC07g23630D	C07	
DEG29	346	3.00E−91	87.00%	BnaA03g40170D	A03	
				BnaA10g23990D	A10	
				BnaC09g48610D	C09	
DEG34	922	0.00E+00	98.00%	BnaA06g40690D	A06	AT4G39270
				BnaC07g47290D	C07	
DEG36	401	4.00E−108	100.00%	BnaA06g30700D	A06	AT3G30260
				BnaA09g02740D	A09	
				BnaC02g38130D	C02	
				BnaC07g25980D	C07	
				BnaC09g02190D	C09	
DEG37	523	1.00E−144	100.00%	BnaA04g26210D	A04	AT2G45470
				BnaA05g04890D	A05	
				BnaC04g04230D	C04	
				BnaC04g50210D	C04	
DEG39	1230	0.00E+00	100.00%	BnaA06g37230D	A06	AT4G38970
				BnaA08g16820D	A08	
				BnaC01g00070D	C01	
				BnaC03g60280D	C03	
				BnaC07g47470D	C07	
DEG41	571	4.00E−159	100.00%	BnaA03g31050D	A03	AT3G08770
				BnaA05g29430D	A05	
				BnaC03g36390D	C03	
				BnaC05g43760D	C05	
DEG42	446	2.00E−121	100.00%	BnaA02g17680D	A02	AT4G30620
				BnaA08g12990D	A08	
				BnaC02g24220D	C02	
				BnaC03g77490D	C03	
DEG43	431	6.00E−117	98.00%	BnaA03g50940D	A03	AT4G34180
				BnaC07g44780D	C07	
DEG44	562	3.00E−156	100.00%	BnaA02g14620D	A02	AT1G69220
				BnaA07g27770D	A07	
				BnaC06g30400D	C06	
DEG46	200	7.00E−48	99.00%	BnaA01g27710D	A01	AT3G16640
				BnaA03g34250D	A03	
				BnaA05g23180D	A05	
				BnaC01g35230D	C01	
				BnaC03g39720D	C03	
				BnaC05g36580D	C05	

DEG, different expression genes.

Subsequently, genes that regulate apical meristem were conducted for further analysis. DEG1 is homologous to the *Arabidopsis* gene *AT4G29040* (*RPT2A*), and it had five copies distributed on A01 (*BnaA01g07860D*), A08 (*BnaA08g13550D*), C01 (*BnaC01g09460D*), C07 (BnaC07g41720D), and C08 (*BnaC08g13300D*). DEG2 had two copies found on A10 (*BnaA10g29350D*) and C09 (*BnaC09g33500D*) and are homologous to the *Arabidopsis* gene *AT5G57950* (*halted root*, *HLR*). DEG4 (*BnaA06g24200D*) is homologous to the *Arabidopsis* gene *AT4G01710* (*crooked*, *CRK*) and encodes ARP2/3 complex 16-kDa subunit. DEG5 had one copy on A01 (*BnaA01g02910D*), which is homologous to the *Arabidopsis* gene *AT4G34220*, and it was considered to be a leucine-rich repeat receptor-like protein kinase gene (*LRR-RLK*). DEG34 had two copies located on A06 (*BnaA06g40690D*) and C07 (*BnaC07g47290D*), and they were homologous to the *Arabidopsis* gene *AT4G39270*, which has the same function with DEG5 and was also considered to be leucine-rich repeat receptor-like protein kinase family gene (*LRR-RLK*). DEG36 is homologous to the *Arabidopsis* gene *AT3G30260* (*AGAMOUS-like MADS-box protein 79*, *AGL79*), which has five copies found on A06 (*BnaA06g30700D*), A09 (*BnaA09g02740D*), C02 (*BnaC02g38130D*), C07 (*BnaC07g25980D*), and C09 (*BnaC09g02190D*). DEG46 (*BnaC03g39720D*) is homologous to the *Arabidopsis* gene *AT3G16640* (*TCTP*), which is mainly expressed in meristematic regions of the shoot and root.

### Relative Expression Level of Candidate Genes Based on QTL Mapping and Gene-Fishing

**Figure 8:**
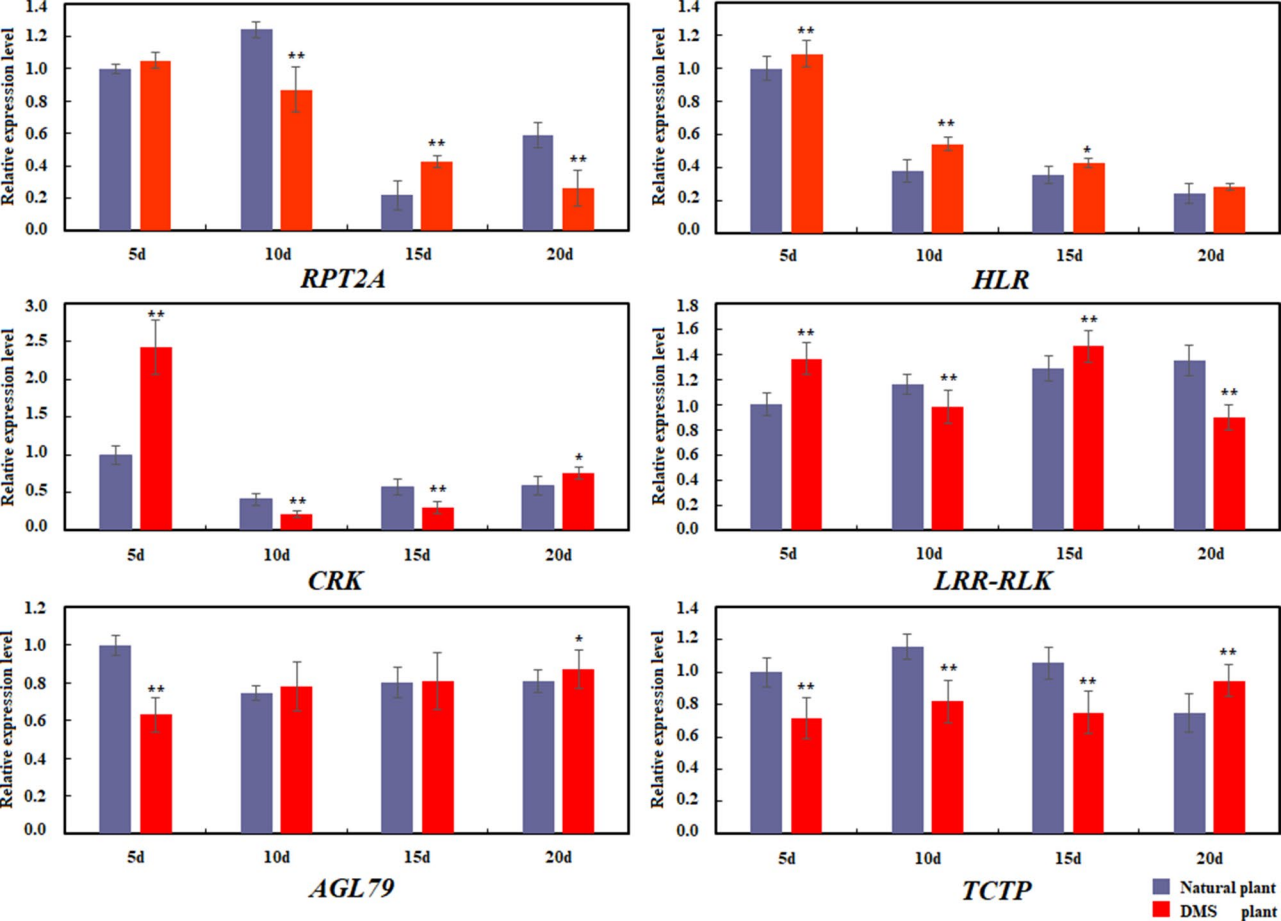
Differential expression of six common genes in natural plant and double-main stem plant. qRT-PCR validation of cloning results of six predictive genes. Left, natural plant (natural). Right, the double-main stem plant (DMS plant). **Significant at p < 0.01. *Significant at p < 0.05. No significance p > 0.05.

Based on the common candidate genes that were obtained from QTL mapping and Gene-Fishing technique, six commonly identified genes were annotated to be related to the formation of MMS, including *RPT2A*, *HLR*, *CRK*, *LRR-RLK*, *AGL79*, and *TCTP*, and these genes were further used to perform the relative expression analysis by quantitative real-time PCR between the normal plant and the DMS plant in four stages of seedling (5, 10, 15, and 20 days of seedling age) (**Figure 8**). It was revealed that the expression level of *RPT2A* was extremely and significantly higher in DMS plant than that of in natural plant in 15 days of seedling age, and in 10 and 20 days of seedling age, the expression level of *RPT2A* in DMS plant was extremely and significantly lower than that of in natural plant. For *HLR*, the expression level in DMS plant was much higher than that in natural plant in all four states of seedling age. Especially in 5, 10, and 15 days, the expression levels in DMS plant were significant or extremely and significantly higher than that in natural plant. In 10 to 20 days of seedling age, expression level were higher in natural plant than that in DMS plant, which were also consistent with Gene-Fishing results (**Table 5**). In addition, whether in natural plant or the DMS plant, expression of *HLR* also showed downward trend in all four states. For *CRK*, in 5 days of seedling age, expression level in DMS plant was extremely and significantly higher than that in natural plant; however, in 10 days of seedling age, the expression of *CRK* was severely inhibited in DMS plant. From 15 to 20 days, expressions of *CRK* were slowly rising again. The decline in gene expression of *CRK* might lead to a decrease in the activity of some regulated genes, which disrupted the balance of SAM. For *LRR-RLK*, the expression level in DMS plant was extremely and significantly lower than in natural plant of 10 and 20 days of seedling age; however, its expression level in DMS plant was extremely and significantly higher than in natural plant in 5 and 15 days of seedling age. In addition, the expression level of *LRR-RLK* in natural plant was the highest at fourth stage, which was compatible with result of Gene-Fishing (**Table 5**). For *AGL79*, the expression level in DMS plant was extremely and significantly lower than in natural plant in 5 days of seedling age. With the development of seedlings, the expression level in DMS plant was slightly higher (not significant) than in natural plant of 10 to 20 days of seedling age. For *TCTP*, the expression level in DMS was extremely and significantly lower than in natural plant of 5 to 15 days of seedling age. In 20 days of seedling age, the expression level of *TCTP* showed upward trend in DMS plant. Relative expression level of candidate genes showed that these six genes had different expression profile between the natural plant and the DMS plant in different seedling age stages.

**Table 5:** Six differential expression gene description and differential expressed in natural plant and the double-main stem plant based on Gene-Fishing.

DEG number	Description	Up expression
DEG1	RPA2A, *Brassica napus* 19S proteasome regulatory subunit 4 homolog A-like.	Natural
DEG2	HLR, *Brassica napus* 26S proteasome non-ATPase regulatory subunit 9-like.	DMS
DEG4	CRK, *Brassica napus* actin-related protein 2/3 complex subunit 5A-like.	DMS
DEG5	LRR-RLK, *Brassica napus* receptor protein kinase-like protein At4g34220.	Natural
DEG36	AGL79, *Brassica napus* uncharacterized LOC106436879.	DMS
DEG46	TCTP, *Brassica napus* translationally controlled tumor protein	DMS

DEG, different expression genes; natural, natural plant; DMS, double-main plant.

## Discussion

Rapeseed is an important oilseed crops worldwide (Cai et al., 2014). Although oil content and seed yield of rapeseed varieties have been effectively improved by rapeseed breeders (Zhao et al., 2011), the optimal plant architectural cultivated varieties are urgently needed for mechanized harvesting and high-density planting in the current agricultural management (Fu, 2008). Rapeseed plant architecture is characterized by plant height, branch number and angles, and inflorescence morphology (Li et al., 2018a), which indirectly influence rapeseed yield by affecting the major yield-component trait (Qiu et al., 2006; Zhou et al., 2014). Crop breeding has primarily focused on plant architecture, including plant height, branch or tiller number and angle, leaf shape, and size (Wang et al., 2018). MMS differentiated from shoot meristem, which is also one of the important factors for improving plant architecture and is conducive to increasing plant density and enhancing seed yield in rapeseed. In KN DH population, several MMS plants were found, and their main characteristics were composed of double- or three main stems from the base of a plant and had less lateral branches (**Figure 1**). Because of nutrient diversion, the MMS plant had slight lower plant height and stronger stem; thus, the MMS materials had stronger lodging resistance. In addition, under a small sowing amount of seeds, the multiple main stem materials not only can effectively increase plant density and the total number of siliques per unit, which finally might increase seed yield, but also, it is suitable for mechanized harvesting and to improve production efficiency. In total, the acquisition of the MMS material provided completely new ideas to improve the plant architecture and breed new varieties in crops.

According to the statistics of the number for MMS materials in different plant environments in several years, MMS was a complex quantitative trait and was significantly influenced by the environment. QTL mapping is considered to be an effective approach for dissecting quantitative traits (Paran and Zamir, 2003). In the present study, 43 QTLs for MMS that were located on 14 chromosomes were identified, explaining 2.95–14.9% of the phenotypic variation (**Table 2**). Two environment-stable QTLs, including *qMMS.A3-2* and *qMMS.C3-5*, were expressed in both winter and semi-winter environments. More importantly, according to epistatic interaction analysis, QTL *cqMMS.C3-5* had three epistatic interactions with other loci, which indicated that this stable QTL was an important loci for MMS. Several environment-stable QTLs for other agricultural traits in rapeseed have been reported, such as stable QTLs for flowering time (Li et al., 2018b; Shi et al., 2009) and thousand seed weight (Zhao et al., 2016). These environment-stable QTLs are suitable for developing new varieties of rapeseed with broad adaptability (Li et al., 2018b). Some genes within QTLs related to cell division and differentiation of shoot meristem were identified mainly included *STM*, *WUS*-related homeobox genes, cytokinin response factors, and auxin-related genes. Previous studies showed that cytokinin (CK) produced by root can promote cell division as well as maintenance of meristem, and the upward transport of CK in the root promotes axillary bud germination and the formation of lateral branches (Chatfield et al., 2000; Leyser, 2003; Ongaro and Leyser, 2008).

Gene-fishing technology has been widely used to identify DEGs in crops (Sanghoon et al., 2009; Liao et al., 2015). Some important differently expressed genes have been identified, such as salt-stress-induced up-regulated genes in barley leaves (Sanghoon et al., 2009) and pistillody genes in wheat (Liao et al., 2015). In the present study, 31 differentially expressed genes between DMS plants and the natural plants of *B. napus* were identified, in which six DEGs involved in the formation and regulation of DMS phenotype (**Table 4**), including *RPT2A*, *HLR*, *CRK*, *LRR-RLK*, *AGL79* (*AGAMOUS-LIKE 79*), and *TCTP* (*translationally controlled tumor protein*). Among them, *RPT2A* is a 26S proteasome subunit and was located in QTL regions of *cqMMS.A1-3*, *cqMMS.A3-1*, *cqMMS.C5-2*, and *cqMMS.C7-3* and was reported to be involved in maintenance of the post-embryonic meristems (Ueda et al., 2004). 26S proteasome is an important factor in signal transduction–mediated pathway; missing or mutation of 26S proteasome would lead to a decrease in strigolactones (SL) content, thereby promoting the excessive growth of axillary buds and other organs, such as branches and tillers (Wang and Bouwmeester, 2018). Therefore, changes of 26S proteasome may affect the growth of SAM and result in MMS phenotype. Abnormal expression of *RPT2A* in DMS plant might change the state of meristem and SL content in 5 to 20 days of seedling age, thereby forming MMS phenotype. *HLR* is located in QTL regions of *cqMMS.A10-1* and *cqMMS.C9-1*, which encodes 26S proteasome regulatory subunit *RPT2A*, which is essential to maintain the size of SAM and might affect the growth of SAM by affecting the expression of *WUS* (Ueda et al., 2004). However, the expression level of *HLR* in DMS plant was much higher than that in natural plant in four states of seedling age, and overexpression of *HLR* might disturb the normal state of meristem and initiate bud differentiation. Meanwhile, *HLR* is also required to regulate the cell division pattern in SAM. In plants, the actin cytoskeleton is essential for various processes such as stomatal closure, cell proliferation, and cell morphogenesis (Akin and Zipursky, 2014). *CRK* encodes ARP2/3 complex 16-kDa subunit. In moss *Physcomitrella*, when the constituent part arpc4 of the *CRK* is lost, shoot growth of the apical cell slows down obviously. In the current study, *CRK* was located in QTL region of *cqMMS.A6-1*, and the decline in gene expression of *CRK* might lead to a decrease in the activity of some regulate genes in seedling. Studies in *A. thaliana*, when the *CRK* is absent, the actin of trichomes, hypocotyls cells, and epidermal pavement cells gather into bundles, downstream cell activity was interfered (Deeks and Hussey, 2003; Quatrano et al., 2006), which disrupted the balance of SAM. *LRR-RLK* represented a large family of protein kinases; it was located in QTL region of *cqMMS.A1-2* (Gou et al., 2010). Expression of *LRR-RLK* had upward trend in DMS plant, which indicated that *LRR-RLK* might participate in the occurrence of axillary buds (Yaginuma et al., 2011). In addition, *LRR-RLK* contains* ERECTA* (*ER*) and *CLV1*. *ER*-family receptor kinases can regulate stem cell homeostasis to regulate organ shape and inflorescence architecture *via* cushioning its CK response in the SAM (Shchennikova et al., 2017). *CLV1* interacts with the *WUS* gene to maintain the balance of differentiation of shoot and floral meristem cells in SAM (Gou et al., 2010). When CK stimulates the expression of *WUS*, the expression of *CLV3* should have been inhibited to result in a SAM expansion; however, the morphology of SAM showed no difference with the normal plant. These phenomena could owe to the cushioning mechanism of ER family, which prevents the expression of *CLV3* from increasing remarkably, thereby maintaining the normal development of stem meristem (Torii et al., 1996; Uchida et al., 2013). *AGL79* is a transcription factor; relatively lower *AGL79* overexpression would promote more lateral branches and rosette leaves (Gao et al., 2018). In addition, miR156/SPL10 modulated lateral root development and branching and leaf morphology in *A. thaliana* by silencing *AGL79* (Gao et al., 2018). *TCTP* is a central regulator of cell proliferation and differentiation in animals and in plants, which is an important mitotic regulator (Amson et al., 2013; Roberto et al., 2015).

Furthermore, some genes also have similar functions in other crops. *HLR* gene is a homolog of the 26S proteasome subunit, which was strongly expressed both in the SAM and RAM of rice seedlings (Yanagawa et al., 2002). *LRR-RLKs* play an important role in regulating the SAM and microspores (Becraft, 2002), brassinosteroid perception, and floral abscission (Carles and Fletcher, 2003; Li, 2003). Rameneni *et al*. performed the whole-genome sequencing (WGS) of *B. rapa* and identified the *LRR-RLK* of *B. rapa*, which is involved in the plant morphological characters and plant stress resistance (Rameneni et al., 2015). *ERECTA* is one of *LRR-RLK* family, which coordinates *Arabidopsis* organ growth and flower development by promoting cell proliferation (Torii et al., 1996; Shpak et al., 2004). *VEGETATIVE1* (*VEG1*) is an AGL79-like MADs-box gene; the secondary inﬂorescences in nutation were replaced by vegetative branches in pea (Berbel et al., 2012). In addition, *AGL79* is involved in regulating leaf shape, shoot branching, and flowering time in *A. thaliana* (Gao et al., 2018). In addition, when maize is under stress response, *TCTP* expression showed a significant upward trend (Chen et al., 2014). Taken together, a series of balance will be broken when these genes mutated and may lead to disturbance in the growth of SAM and form abnormal organ morphology, such as clumping leaves and multiple axillary buds (Gou et al., 2010; Uchida et al., 2013).

## Conclusion

The increase of yield and oil content is the ultimate goal in rapeseed breeding. Due to the lack of mechanized cultivated variety and high productive cost as well as low income, farmers prefer to idle the farmland rather than plant rapeseed, which seriously hampers the development of rapeseed in China. Importantly, the acquisition of MMS materials provides the possibility for breeding mechanized varieties, high-density planting varieties, and the yield improvement in rapeseed. In the present study, six common candidate genes within QTL regions and differentially expressed genes, including *RPT2A*, *HLR*, *CRK*, *LRR-RLK*, *AGL79*, and *TCTP* were identified by using QTL mapping and Gene-Fishing technique. Meanwhile, according to their functional annotation, these candidate genes might be related to the formation of MMS phenotype, in which genes might disrupt cell division and the differentiation of SAM and induce the formation of multiple buds, thereby promoting the formation and development of the MMS plant. MMS is an important agriculture trait, which is differentiated from shoot apical meristem. Relative expression level of candidate genes showed that these genes had different expression level between the natural plants and the DMS plants in different seedling age stages. Overall, the study would not only give a new insight into the genetic basis underlying the control of the MMS in rapeseed but also provide clues for plant architecture breeding in rapeseed.

## Data Availability

All datasets generated for this study are included in the manuscript/**Supplementary Files**.

## Ethics Statement

The authors declare that the experiments comply with the current laws of the country in which they were performed.

## Author Contributions

WZ wrote the first draft of the manuscript. WZ and HC conducted all the field experiments, performed most of the experiments and data analysis for the overall study; HC, LnZ, NT, YZ, BL, KZ, ZG and HW helped and participated in the field trials and data collection for multiple-main stem. KC, LbZ and DH performed the phenotypic data processing and QTL detection. ML designed and conceived the overall study and revised the manuscript. All authors read and approved the final manuscript.

## Funding

This work was supported financially by the national key research project of China (2016YFD0101300, 2016YFD0101200), the Science and Technology Plan Project of Yangling Demonstration Zone of China (2017NY-20), Innovative Talents Promotion Plan of Shaanxi province- Key Technological Innovation Team Plan (2017KCT-23).

## Conflict of Interest Statement

The authors declare that the research was conducted in the absence of any commercial or financial relationships that could be construed as a potential conflict of interest.

## Abbreviations

*B. napus*, *Brassica napus*; *A. thaliana*, *Arabidopsis thaliana*; QTL, quantitative trait locus; SNPs, single nucleotide polymorphisms; DL, Dali in Shannxi Province, China; WH, Wuhan in Hubei Province, China; MMS, multiple main stem; DMS, double-main stem; DEGs, different expression genes; DH, doubled haploid; qPCR, quantitative real-time PCR.

## Supplementary Material

Figure S1 Differential expressed genes screen using ACPs amplified primer 1-9. Odd number lane represented natural plant and even number lane represented double-main stem plant.

Figure S2 Differential expressed genes screen using ACPs amplified primer 10-15. Odd number lane represented natural plant and even number lane represented double-main stem plant.

Figure S3 Differential expressed genes screen using ACPs amplified primer 16-20. Odd number lane represented natural plant and even number lane represented double-main stem plant.

Figure S4 Differential expressed genes screen using ACPs amplified primer 21-25. Odd number lane represented natural plant and even number lane represented double-main stem plant.

Figure S5 Differential expressed genes screen using ACPs amplified primer 26-31. Odd number lane represented natural plant and even number lane represented double-main stem plant.

Figure S6 Differential expressed genes screen using ACPs amplified primer 32-36. Odd number lane represented natural plant and even number lane represented double-main stem plant.

Figure S7 Distribution of QTLs for multi-main stem on A genome in B. napus

Figure S8 Distribution of QTLs for multi-main stem on C genome in B. napus
