# Supplementary figures and images for: Integration of QTL Mapping and Gene Fishing Techniques to Dissect the Multi-Main Stem Trait in Rapeseed (Brassica napus L.)
Source: Front Plant Sci. 2019 Sep 20;10:1152. doi: 10.3389/fpls.2019.01152 (PMC6764107; doi:10.3389/fpls.2019.01152)

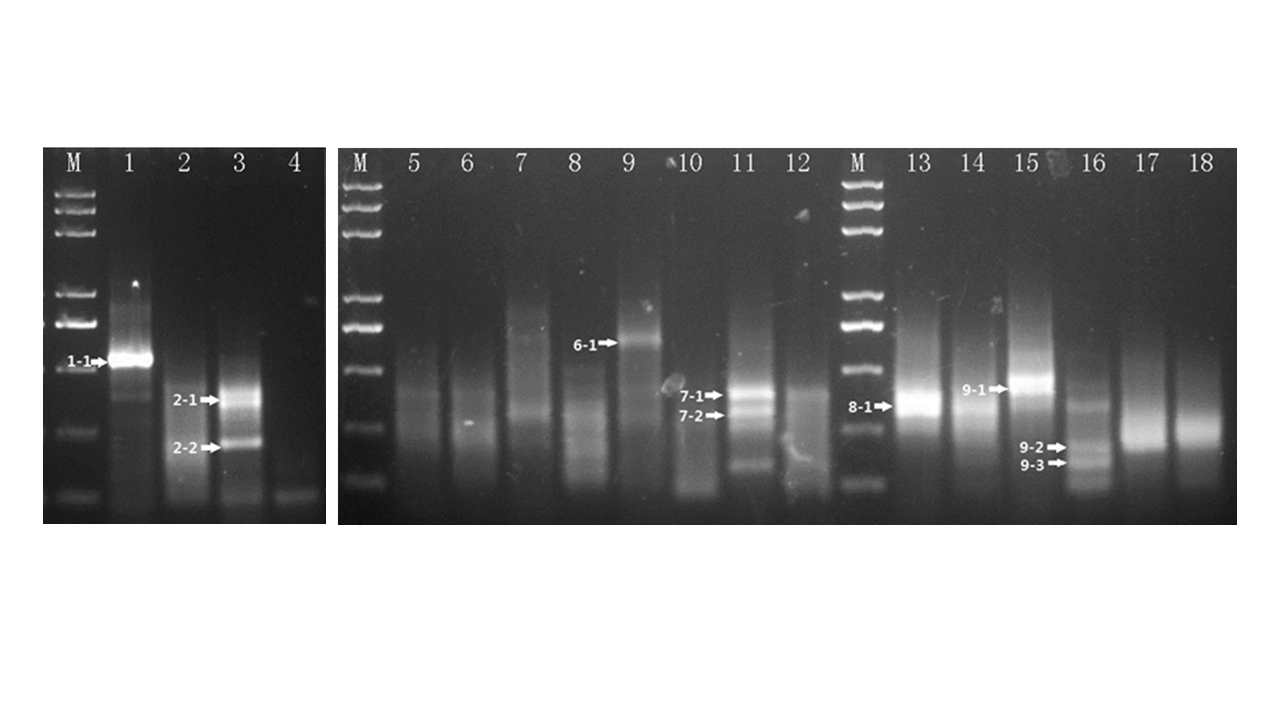

Supplement: Figure S1 — Differential expressed genes screen using ACPs amplified primer 1-9. Odd number lane represented natural plant and even number lane represented double-main stem plant. [file Image_1.tif]

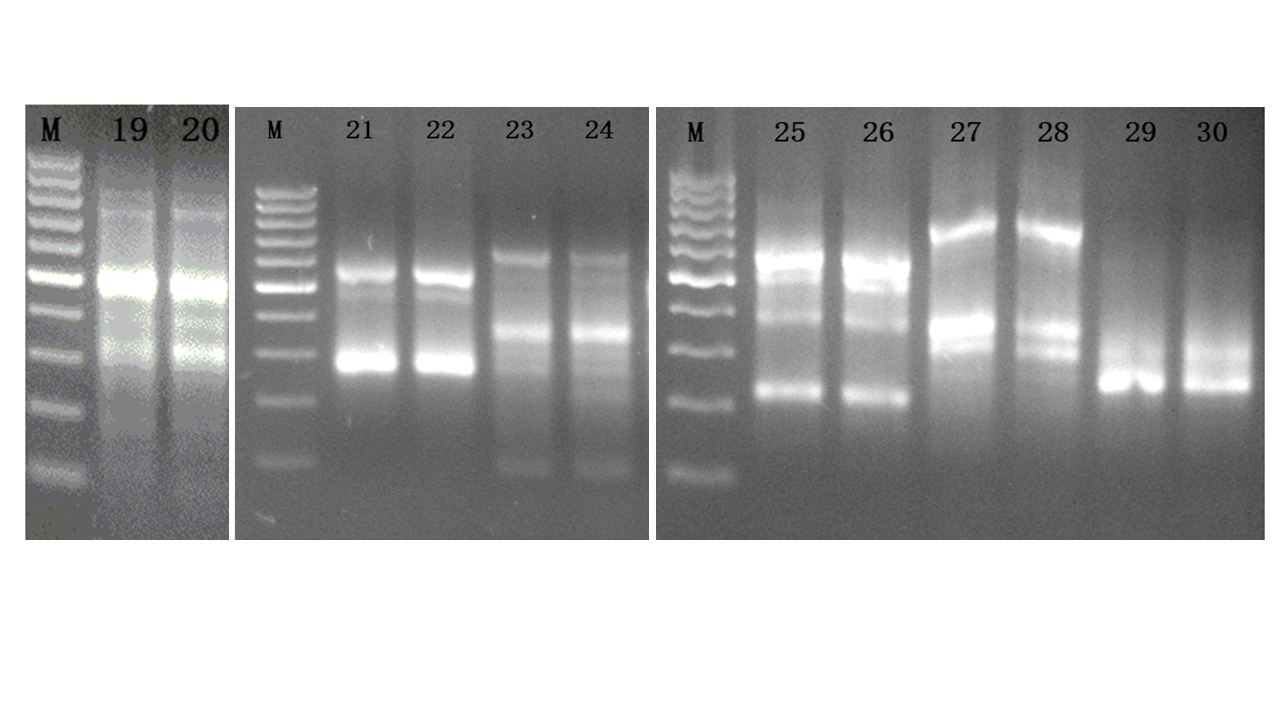

Supplement: Figure S2 — Differential expressed genes screen using ACPs amplified primer 10-15. Odd number lane represented natural plant and even number lane represented double-main stem plant. [file Image_2.tif]

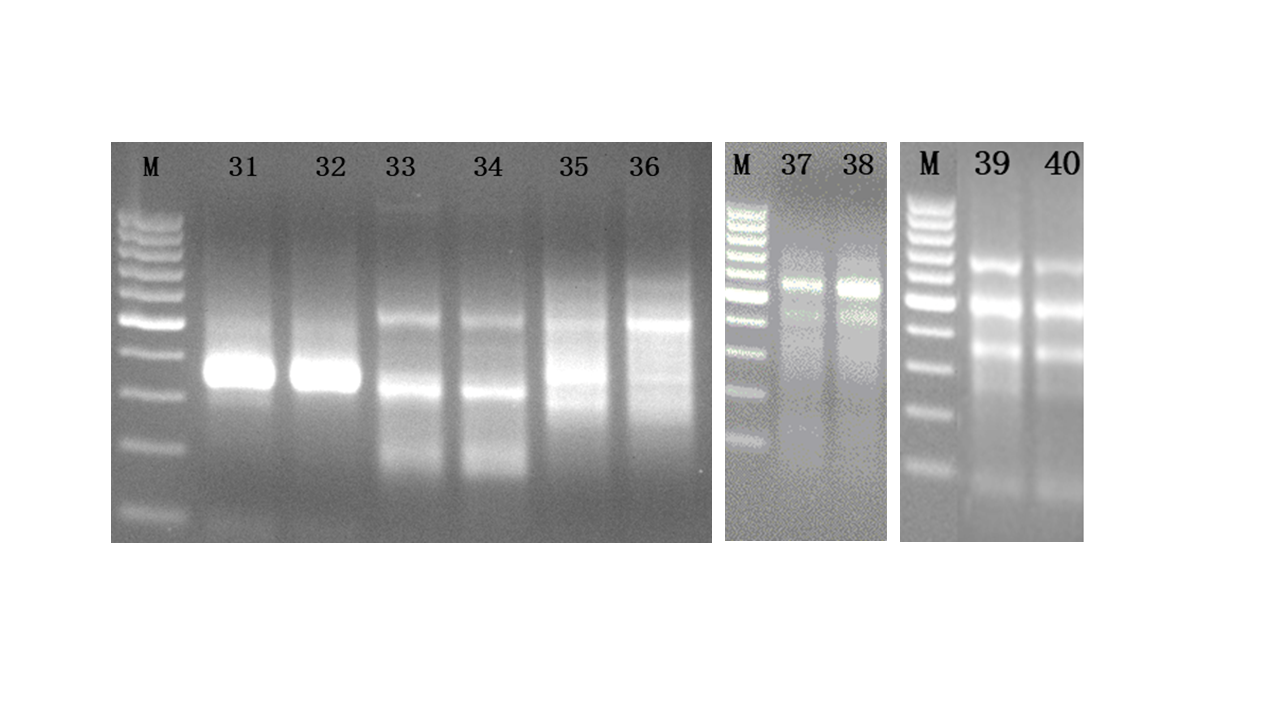

Supplement: Figure S3 — Differential expressed genes screen using ACPs amplified primer 16-20. Odd number lane represented natural plant and even number lane represented double-main stem plant. [file Image_3.tif]

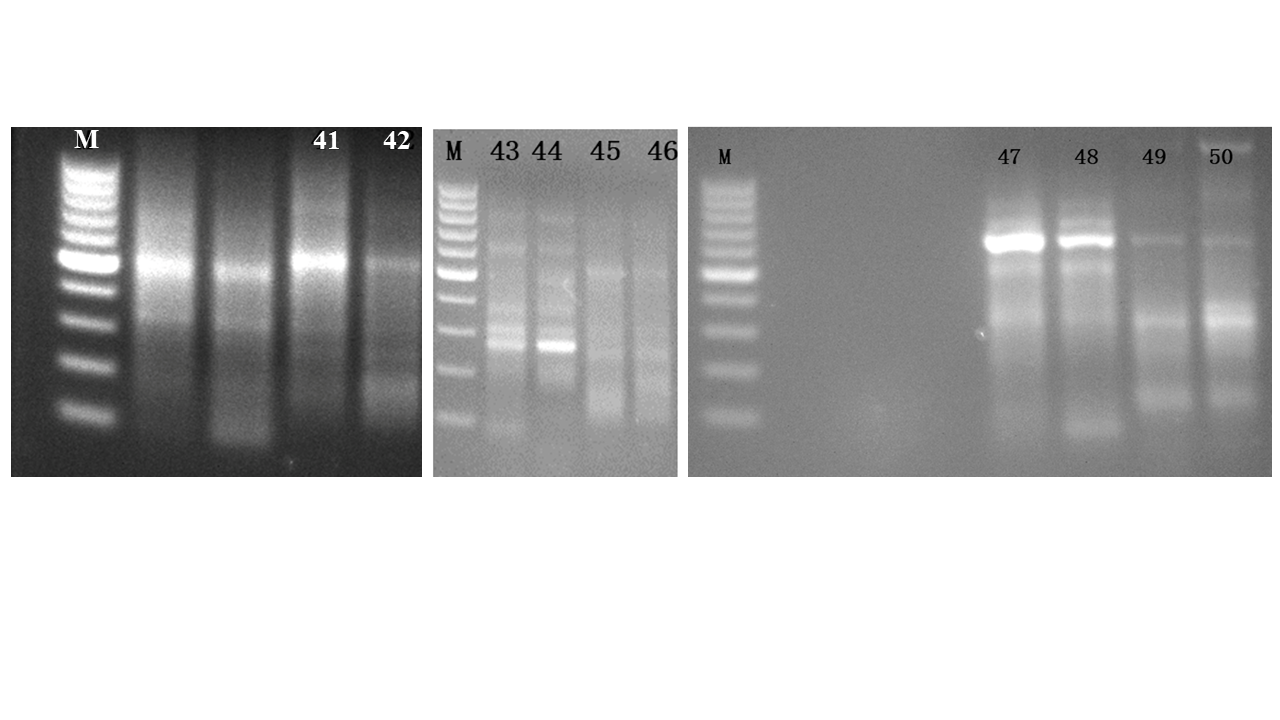

Supplement: Figure S4 — Differential expressed genes screen using ACPs amplified primer 21-25. Odd number lane represented natural plant and even number lane represented double-main stem plant. [file Image_4.tif]

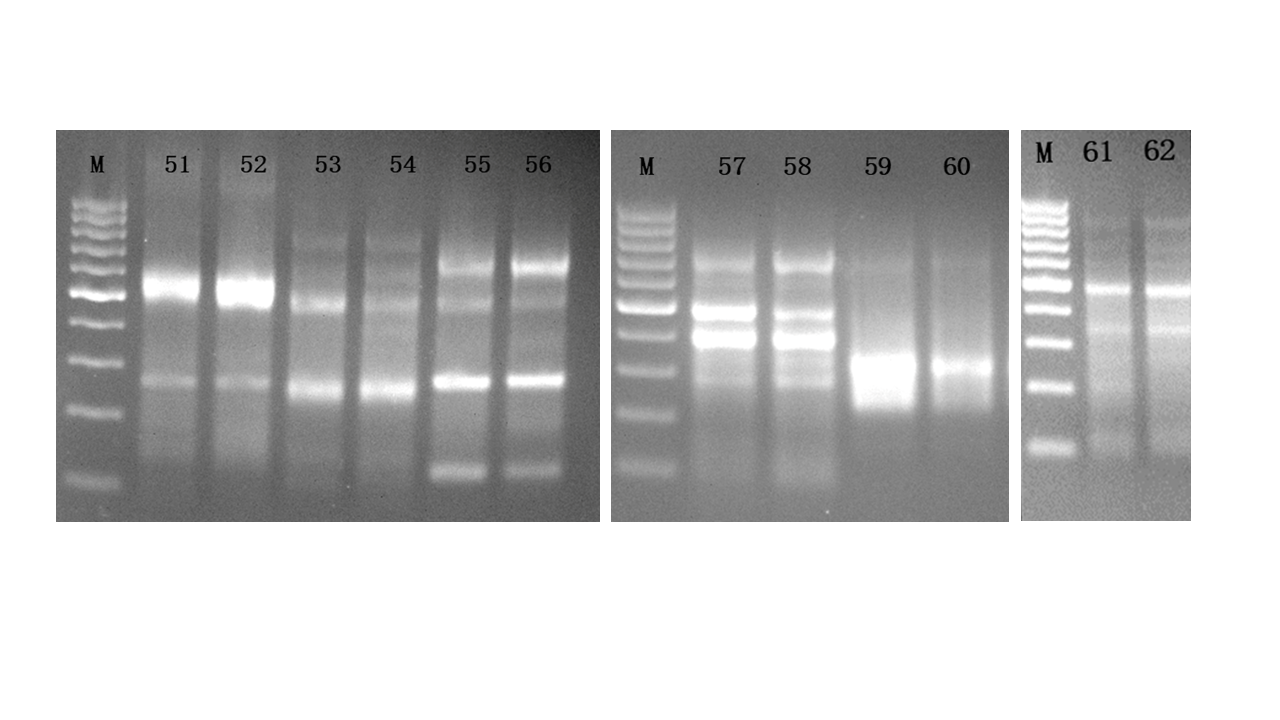

Supplement: Figure S5 — Differential expressed genes screen using ACPs amplified primer 26-31. Odd number lane represented natural plant and even number lane represented double-main stem plant. [file Image_5.tif]

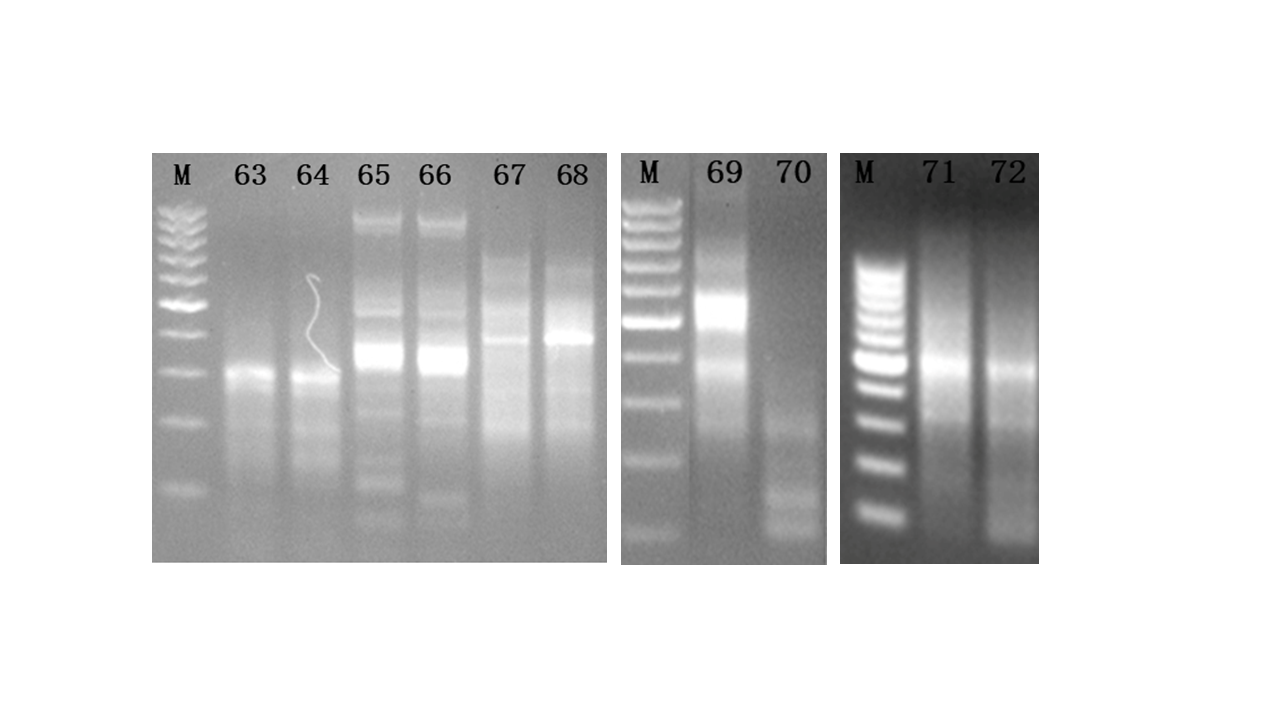

Supplement: Figure S6 — Differential expressed genes screen using ACPs amplified primer 32-36. Odd number lane represented natural plant and even number lane represented double-main stem plant. [file Image_6.tif]

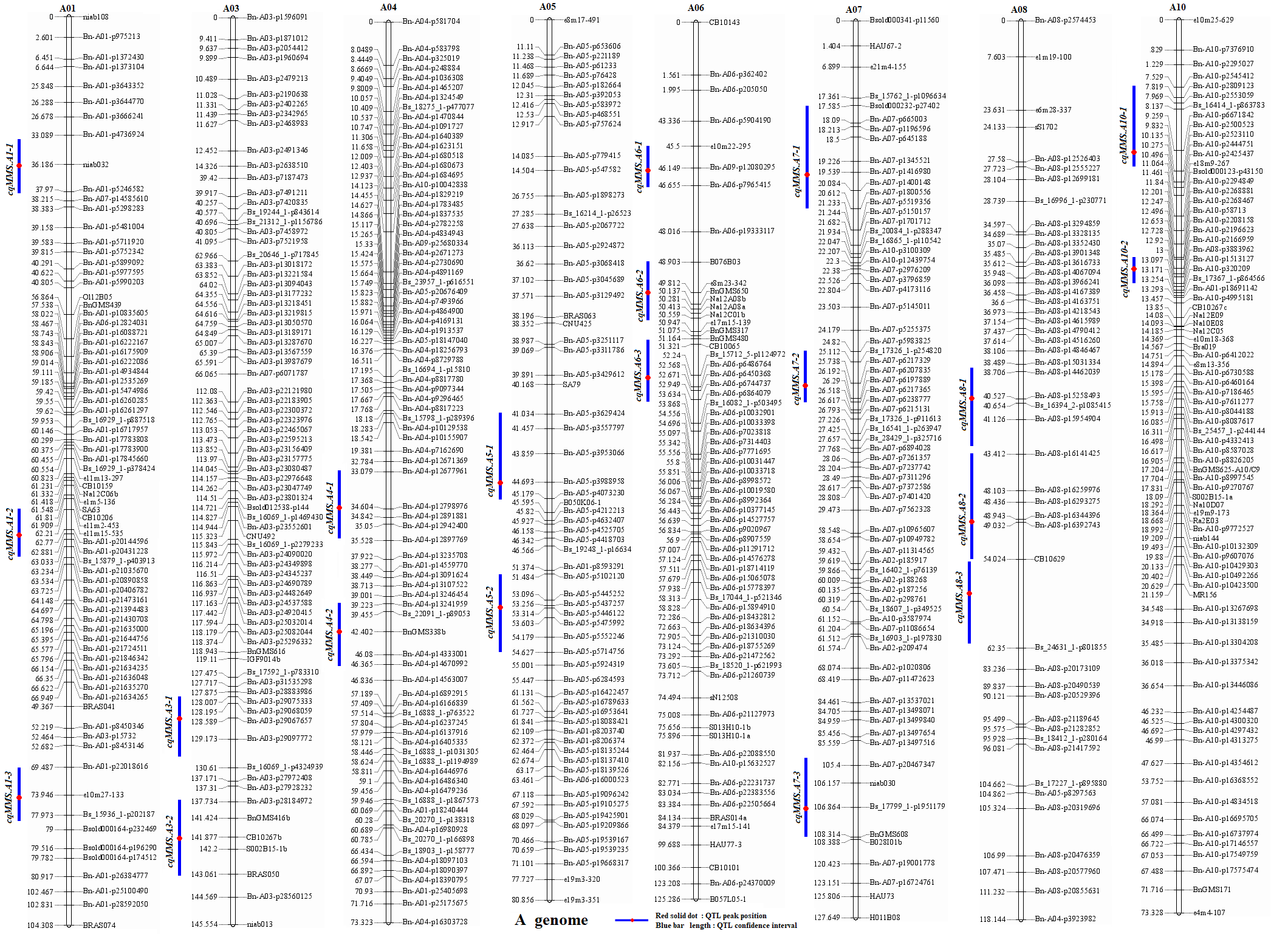

Supplement: Figure S7 — Distribution of QTLs for multi-main stem on A genome in B. napus [file Image_7.tif]

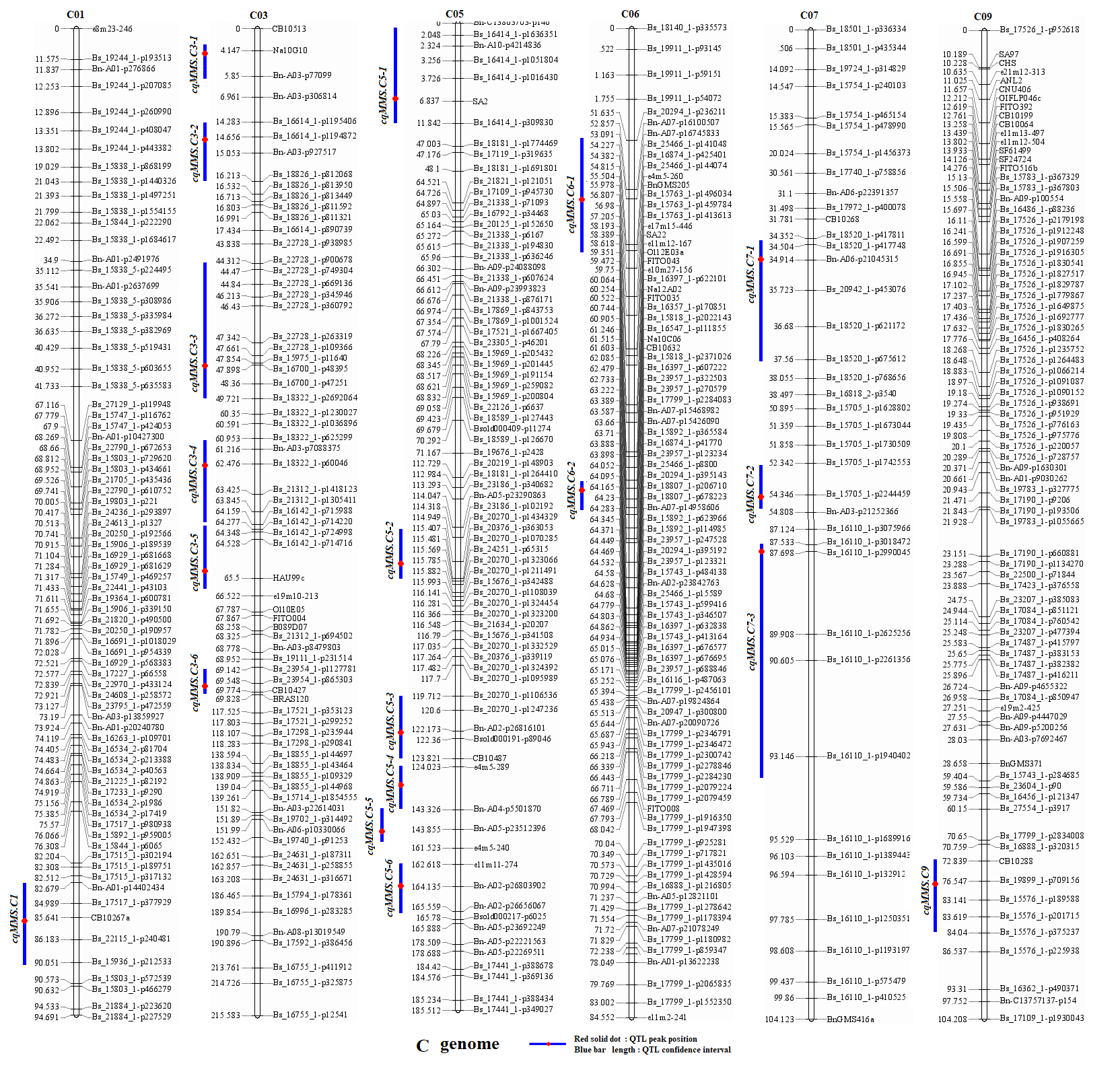

Supplement: Figure S8 — Distribution of QTLs for multi-main stem on C genome in B. napus [file Image_8.tif]
